# Crude Oil Yield Estimation: Recent Advances and Technological Progress in the Oil Refining Industry

**DOI:** 10.3390/s25175511

**Published:** 2025-09-04

**Authors:** Wan Nazihah Liyana Wan Jusoh, Madiah Binti Omar, Abdul Sami, Kishore Bingi, Rosdiazli Ibrahim

**Affiliations:** 1 Department of Chemical Engineering, Universiti Teknologi PETRONAS, Seri Iskandar 32610, Malaysia; abdul_22000064@utp.edu.my; 2 Department of Integrated Engineering, Universiti Teknologi PETRONAS, Seri Iskandar 32610, Malaysia; madiah.omar@utp.edu.my; 3 Department of Electrical and Electronics Engineering, Universiti Teknologi PETRONAS, Seri Iskandar 32610, Malaysia; bingi.kishore@utp.edu.my (K.B.); rosdiazli@utp.edu.my (R.I.)

**Keywords:** yield estimation, crude oil, refining, laboratory analysis, simulation, modelling, machine learning

## Abstract

Oil refineries depend greatly on the estimation of crude oil properties in order to understand the oil’s behaviour and the product fractions expected from the refining process. In yield estimation, the crude oil source and variant can cause variability in prediction and lead to the need for repeatable analysis. The necessity for fast, accurate, and high-generalization yield estimation initiates the framework of this review. This paper aims to comprehensively review the available techniques for estimating the yield of petroleum products in the petroleum refining industry. The review provides a brief overview of petroleum refining processes and high-value products, followed by a description of the traditional method, which utilizes laboratory analysis to offer detailed findings, but requires a tedious methodology. The improvement of yield estimation leads to process simulation, modelling, and machine learning, enabling a fast response and better prediction with higher accuracy. Thorough case studies related to simulation software, models, and algorithms are presented to discover the process and model development, applications, advantages, and drawbacks. Enhancing petroleum product yield estimation provides reliable techniques for oil refiners that enable them to achieve optimized production aligned with sustainability and modernization goals.

## 1. Introduction

Accelerated energy utilization, driven by industrialization and urbanization, has triggered the oil and gas industry to serve as the backbone of global energy providers. The growth of the petrochemical sector has led directly to high feedstock demand, with the oil industry playing a pivotal role in the distribution of its primary resources. Therefore, the oil refining industry requires precise monitoring of process management and control through the implementation of advanced operational strategies, as well as optimization and automation processes. Enhancing process automation enables a reduction in human intervention, simultaneously contributing to lower human error and time consumption. For economic and operational insight, the yield of products and the quality of crude oil are crucial components for ensuring company growth with high profitability [1,2]. The production rate and expected yield can be tailored to meet client demands, enabling accurate profit forecasting.

Furthermore, plant operation and safety can be precisely managed. For example, information on the sulphur content in crude oil can be utilized from an early stage to prevent disruptions in product quality and yield, such as irreversible catalyst poisoning. With respect to optimization insight, yield estimation supports resource optimization and sustainability practices. Converting the traditional methodology of yield prediction to a modern technique greatly improves process efficiency. The implementation of advanced technology, simulations, and artificial intelligence (AI) can have a positive impact on yield by introducing a multi-functional system capable of fast and reliable prediction. The quality and properties of crude oil also influence yield estimation, as its high compositional variety and heavy molecular weight require extensive and controlled analysis [3,4]. Processing conditions also affect yield estimation, as sudden changes in temperature, pressure, or other parameters can cause fluctuations in prediction.

Crude oil yield estimation and product composition vary, which means that multiple tests, some of which are redundant, are required for every single variant to improve prediction accuracy. This situation limits the estimation process and hinders the refinery’s ability to obtain a comprehensive overview of the required information, particularly under timeline constraints. Common practices for yield estimation involve laboratory analysis for a thorough investigation. A single crude oil batch requires multiple tests to gain a comprehensive understanding of its properties and accurately estimate its yield. However, some challenges in yield estimation related to prediction accuracy, analysis cost, maintenance expenditure, and time usage limit the optimization of refinery operations [5]. Process simulation is an innovative approach for oil refineries that reduces sole dependency on the laboratory method and provides a good basis for comparison. Recent technological development has included artificial intelligence (AI)-driven approaches that simultaneously enhance both processes and provide better prediction results.

Although numerous reviews have been published on individual yield estimation methods, to the best of our knowledge, a comprehensive study comparing crude oil yield estimation techniques, including laboratory, simulation, and AI methods, has not been explored. A clear understanding of available technologies can address limitations while aligning with the state of the art in the oil and gas industry. This review paper focuses on methods for yield estimation by applying laboratory analysis, process simulation and machine learning. The remainder of the paper is organized as follows: Section 2 introduces refinery processing units and product fractions, while Section 3 provides a brief introduction to yield estimation methods, including their advantages and disadvantages. Section 4 details laboratory techniques, including gas chromatography, physical and chemical analysis, and spectroscopy. Subsequently, Section 5 emphasizes the process simulation methodology, and Section 6 explains mathematical modelling and machine learning. Finally, Section 7 focuses on a hybrid method that combines laboratory, simulation, and modelling techniques, followed by a discussion of directions for future study.

## 2. Oil Refinery and Petroleum Products

The petroleum industry plays a crucial role in the global economy, with fast-growing industries supported by advancements in its technology. As of 2023, the petroleum industry contributed 32% of global annual gross domestic product (GDP), and specifically in Malaysia, its contribution is up to 20% [6,7]. With the increasing demand for energy, this industry is crucial in supporting the supply chain by providing high-quality products for both industrial and societal use. Nowadays, petroleum refining has expanded to optimize operations, transforming crude oil into high-value products.

**Figure 1:**
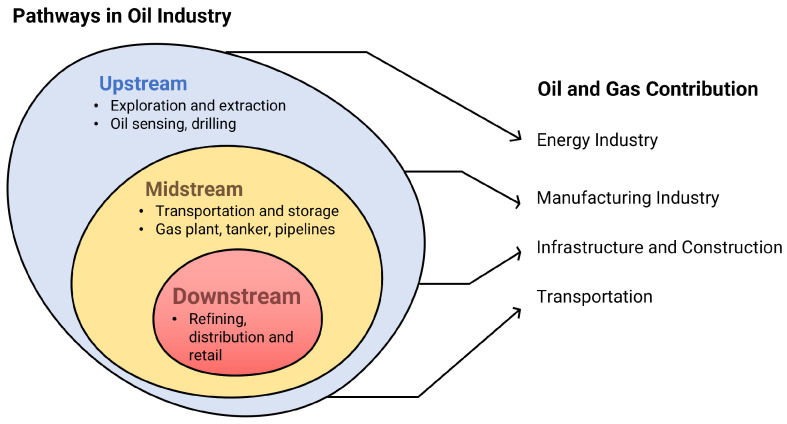
Contribution and pathways in the oil industry [8,9,10].

The petroleum industry is divided into three categories: upstream, midstream, and downstream (Figure 1) [8,9,10]. Upstream focuses on exploration and extraction, relying on technologies that sense oil features, such as surface geological remote sensing, gravity and magnetic data, seismic surveys, and well drilling [11]. The midstream process includes gas plants, LNG tankers, gasification, and oil and gas pipelines. Meanwhile, downstream highlights the refining process of crude oil into specific products. The petroleum industry also supports various sectors, including energy, manufacturing, transportation, and infrastructure, with a focus on providing heating sources, petrochemical feedstock, and fuel-based energy and supporting power generation, machinery maintenance, and road construction.

### 2.1. Oil Refining Process

An oil refinery produces high-value products through several processes, depending on layout and company needs. The primary process refers to rectification involving the separation of crude oil through atmospheric distillation or vacuum distillation [12]. Both techniques separate fractions based on their boiling ranges. The hydrocarbon molecule is exposed to changes in chain structure, specifically the chain length, during the conversion phases [13]. The process consists of decomposition using thermal and catalytic cracking, unification by polymerization and alkylation, and alteration through isomerization and catalytic reforming [14,15]. Treatment processes remove contaminants using physical or chemical processes to prepare finished products. Crude oil is first stored in a storage tank to allow water and sediments to settle [15,16]. It is then subjected to a pretreatment operation involving desalting and dewatering, followed by a water-washing operation at a temperature of 102–138 °C for further cleaning to remove water-soluble minerals, solids, contaminants, and salts [15,17,18].This particular operation is crucial, especially for sour and high-contaminant crude oil, due to the risk of equipment plugging and fouling, pipeline corrosion, and catalyst poisoning.

The process furnace is an important unit that is used to heat crude oil until it reaches the high temperatures required for efficient and economical processing [19]. Preheating increases the temperature to within the range of 343–371 °C; however, excess heat may cause thermal cracking and coke deposits, leading to clogging and shutdowns [20]. The temperature profile must be carefully maintained, and preheated crude oil is usually introduced between the middle and bottom of the crude distillation column [21]. Crude oil contains a mixture of hydrocarbons, which all exhibit different molecular weights, boiling points, and compounds [17].

The atmospheric distillation or crude distillation step focuses on separating the lighter boiling fraction of crude oil. In contrast, vacuum distillation is used to recover the heavier component from the atmospheric residue under reduced pressure. Each separated fraction, or cut, has a defined initial boiling point (IBP) and end boiling point (EBP) to meet the specific requirements of the desired products [20]. This step is carried out using a column whose internal design is made up of plates or trays for cut entrapment and collection; the vapour rises in the column, interacts with cool liquid, and flows down [17]. A side stripping column is also included for refining products using their IBP and EBP [20]. The temperature and pressure highly influence the movement of vapour and liquid inside the column, as the plates and trays provide resistance [22,23]. For vacuum distillation, separation of the heavy component requires steam to prevent thermal cracking, and a column with a larger diameter is used due to the higher volume required for vaporizing the crude oil [20].

**Figure 2:**
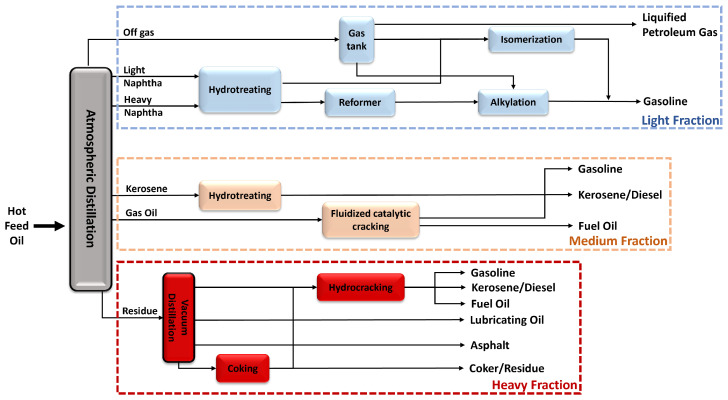
Distillation column process flow diagram for fractionation, conversion, and purification process [15,20].

The various processes involved in crude oil fractionation and conversion are presented in Figure 2. The isomerization and alkylation process primarily yields naphtha and gasoline, while kerosene is obtained directly from its side stripper after being refined in the hydrotreating process. Meanwhile, for gas oil, fluidized catalytic cracking (FCC) fractionates the product yield into gasoline and fuel oil. The hydrocracking process, which is part of vacuum distillation, converts heavier distillates into gasoline, kerosene, diesel, and fuel oil. The conversion process involves multiple methods for transforming heavier products into a lighter composition, known for its higher commercial value. The thermal process consists of thermal cracking, viscosity breaking, and coking, which are the subsequent processes after vacuum distillation, focusing on improving the production of naphtha, gasoline, and kerosene [15,24]. Thermal cracking occurs at 455–540 °C and 689–6895 kPa, and commonly, gas oil is the major product for fuel oil and diesel fuel.

Catalytic processes produce higher-octane products with fewer heavy residues in the presence of catalysts [25]. In this process, activated natural or synthetic material is implemented in the form of beads, pellets, or powder to improve its interaction with the distillate or residue [26,27]. Catalysts should withstand physical impact and have high resistance to carbon dioxide, air, nitrogen, sulphur, and steam. The catalytic reforming process involves applying higher temperatures with the same equipment as used for thermal cracking, and it intentionally reforms low-value hydrocarbon feedstock into a higher-value product [28]. A similar concept is applied in the isomerization process, where normal paraffins are converted into high-octane gasoline with a lower boiling range, and a catalyst is used to reduce the occurrence of side reactions. In the alkylation process, the conversion of unstable olefins by combining them with paraffin results in the formation of higher isoparaffins. The last conversion process is polymerization, which transforms olefin gases into liquid products, such as gasoline, using a catalyst at lower operating temperatures [29].

Another process of conversion is hydroprocessing, which can be divided into several methods, including hydrotreating, hydrorefining, and hydrocracking. Hydrotreating serves as a process for the removal of impurities such as sulphur, nitrogen, salts, olefins, and aromatics, without the intention of changing the boiling range of feedstock [20]. The hydrocracking process is an expensive method due to the need for a hydrogen supply; however, it is capable of producing a high yield of isoparaffins and lighter products, while limiting the production of olefins [20]. Typically, hydrocracking is used for the purification of cycle oils, thermal and coker oils, and heavy cracked and straight-run naphtha, which face difficulties in fluid catalytic cracking (FCC) or reforming processes [15]. Distillates from the conversion process are treated and blended into specific cuts before being used as the final product.

### 2.2. Product Distribution in Oil Refining

**Figure 3:**
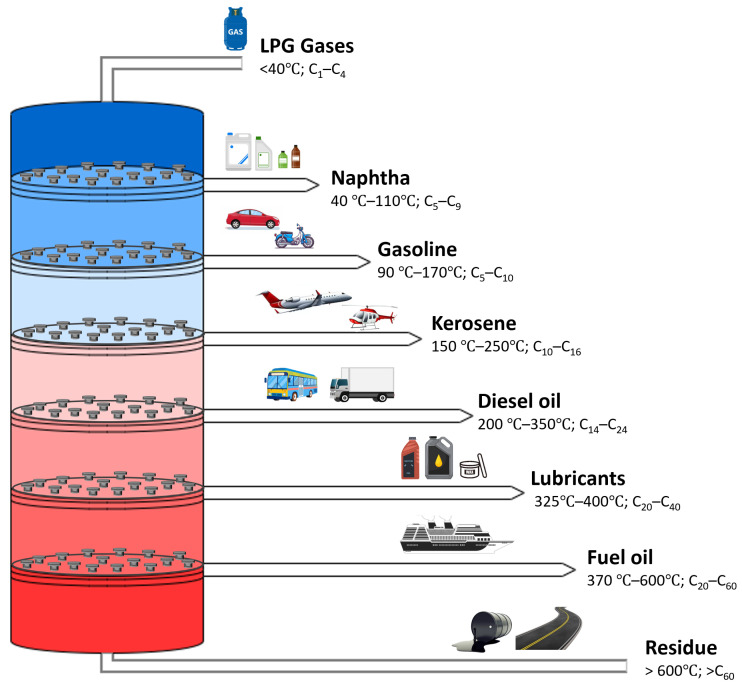
End product fractions.

As mentioned previously, oil refineries draw out various products, with many applications, from the feedstock of the petrochemical industry, including liquified petroleum gas (LPG), aviation fuel, and lubricant, as shown in Figure 3. The products are separated based on their boiling point; the lightest is LPG, with a boiling point below 40 °C, a density of 540 kg/m^3^, and hydrocarbons between C_1_ and C_4_, which are the simplest hydrocarbons [20]. LPG, especially propylene and butylene isomers, is highly used as a chemical feedstock and is available in gaseous form at ambient temperature, while it is liquid under moderate pressure [30,31]. The conversion of gas to liquid LPG can be conducted through mixing, chemical conversion, or cryogenic condensation; the liquid is more convenient and safe for transportation, as well as providing economic benefits [32]. LPG has a very low sulphur content, is easy to ignite, produces clean combustion gas, and is widely used in cooking, refrigeration, and heating applications [33,34,35].

Naphtha is a liquid product of the top distillation column, representing the low–medium boiling fraction [30]. It typically represents 15–30 wt% of crude oil, and it starts to distil at a temperature of 40–110 °C, dividing into light, medium, and heavy fractions with a density of around 650 kg/m^3^ [36]. The quality of naphtha is very high; it lacks the presence of any contaminants such as catalysts and sulphur, as its distillation occurs at a low temperature and in the top column [36]. Regarding its usage, naphtha serves as a precursor for other liquid fuels, as a blend stock, and as a solvent in paints, and is also used in dry cleaning, asphalts, rubber, and industrial extraction processes [30,36,37,38,39]. Gasoline contains a mixture of hydrocarbons (C_5_–C_10_), similarly to naphtha, and has a boiling temperature of 90–170 °C and a density of 700–750 kg/m^3^. Its high volatility and flammability make gasoline suitable for use as an automobile fuel due to its promising capacity for blending and the energy provided as a result of its combustion [30]. Gasoline has been widely used in aviation, jet fuels, machinery, additives, inhibitors (oxidation and corrosion), dyes, and oxygenates [30,40,41,42].

Kerosene, also known as paraffin oil, is a middle distillate product obtained from atmospheric distillation at 150–250 °C in liquid oil form, with a colour that is pale yellow to colourless [30]. Kerosene’s volatility lies between that of gasoline and diesel; it also has an odour and is flammable. Its density is 800 kg/m^3^, and the composition of kerosene usually consists of 10 hydrocarbons from C_10_–C_16_ per molecule and should be free from aromatic, unsaturated hydrocarbon and sulfur [37,43]. Kerosene is universally used in jet fuels, including in both commercial and military contexts, as well having industrial uses as a solvent and for blending, lighting, and heating [44,45,46]. Diesel is produced by blending cuts from distillation, hydrocracking, FCC, visbreaking, and coking to increase the production volume [47]. The boiling point range of diesel fuel is 200–350 °C, and it has a density of 850 kg/m^3^ and a carbon number of C_14_–C_24_, exhibiting similarity with kerosene, which makes some similar analysis applicable for product specification [30,37,48,49,50].

Lubricating oil is a product that results from dewaxing and clay treatment, and is characterized by a boiling point between 325 °C and 400 °C, a density of 890 kg/m^3^, and a hydrocarbon range from C_25_ to C_40_. The addition of additives and viscosity enhancers in the base stock improves the product’s criteria for its usage as a motor oil, grease, and lubricant in engine maintenance [47,51,52,53]. The boiling range of this fuel oil falls within 370–600 °C, it is categorized as distillate and residual, and it has a density of 900–1000 kg/m^3^. The distillate fuel oil has a fixed boiling range and no heavy components, while the residual oil contains some residue [37]. It has a high viscosity, most likely due to the presence of tar balls and emulsions, which causes constraints in pumping and dispersion, leading to the need for blending [47]. It is widely used in heating vessels, power plants, industrial facilities, and heavy transportation [44,54,55,56]. The final product fraction is a residue with a boiling temperature exceeding 600 °C, which is used as bitumen and coke, with densities of 1500–1900 kg/m^3^ and 2100 kg/m^3^, respectively. Bitumen is commonly used in road construction as a binder and for railway tracks; meanwhile, coke is used as an energy source, a carbon electrode, and in pot liners [57,58,59,60,61,62,63].

## 3. Yield Estimation of Petroleum Products

**Figure 4:**
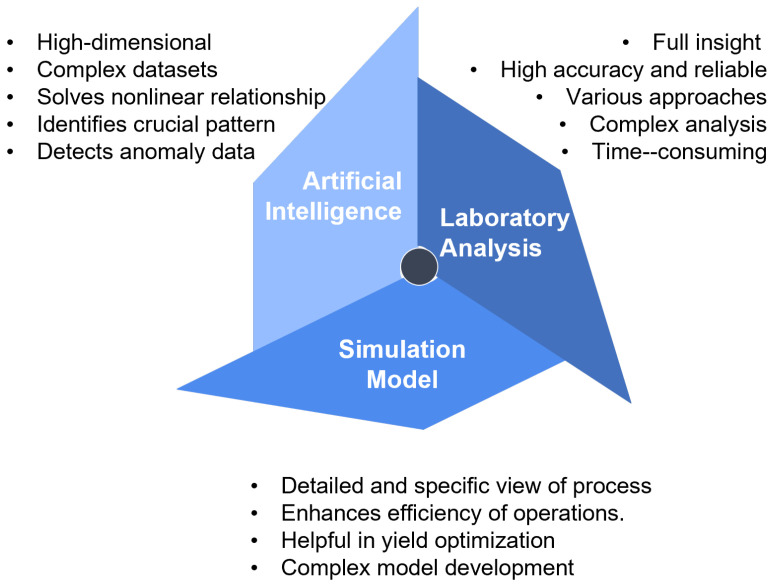
Typically practised yield estimation method.

Yield estimation, in general, refers to the process of predicting the yield obtained from a variant of crude oil received in the oil refinery. Each crude oil has different properties and a different chemical composition, which allows for a variety of product yields and qualities. This estimation process is crucial for providing initial insight into products, including their type, their oil composition, the volume of each product section, and their quality. Crude oil yield prediction can be achieved through several approaches, including laboratory analysis, simulation software, historical data analysis, and machine learning. Figure 4 shows the product yield estimation process that is commonly practiced in the oil refinery, and a detailed discussion is provided in the next section. In general, laboratory analysis provides a reliable method that offers complete insight into product properties and facilitates yield estimation through multiple tests. However, it tends to be too complex and requires a longer analysis period. The simulation modelling technique enables a detailed and specific view with high efficiency, in addition to the standard practice for yield optimization. However, model development is more complex and requires detailed information for high prediction accuracy. Yield prediction using an artificial intelligence method serves the purpose of handling high-dimensional, complex datasets and solving nonlinear relationships through the development of a model pattern and data training, but requires large datasets for model generalization.

The information obtained from predictions is helpful to the oil refiner, as it may influence the economic outlook of the product and ensure sales and market alignment. Refineries can select suitable crude oil and products according to market demand. The petroleum refinery can use the estimated yield value obtained as initial data to propose a product price to the targeted client. Additionally, yield estimation is also helpful in determining the process requirements and optimizing the refining of crude oil by taking advantage of its physical and chemical properties. Some crude oils may have a high content of water and sediments, requiring a longer sedimentation process for complete separation. Furthermore, sour crude oil, which has high sulphur content, must undergo a dewatering process to remove its water content. This additional process and more prolonged crude oil pretreatment directly impacts the operational cost and production time. Predicting the yield of petroleum products ensures regulatory compliance and enables the products to meet fuel standards.

## 4. Laboratory Techniques

Laboratory analysis, such as crude assay data analysis, is a typical and conventional method for determining the properties, composition, and yield estimation of crude oil. The crude test assay provides a detailed and accurate description of the oil’s behaviour during refining and the quality of the end products. Furthermore, the presence of contaminants, which can potentially increase processing costs, can be eliminated. The crude test assay is divided into two categories: the inspection assay and the comprehensive assay. The inspection assay is routinely conducted on all crude oil received in the refinery before processing. If a significant difference is found in the assay data compared to historical data, a more comprehensive assay test will be conducted. The comprehensive assay provides a very detailed assessment, but involves analyses that require a lot of time and high costs [64].

**Figure 5:**
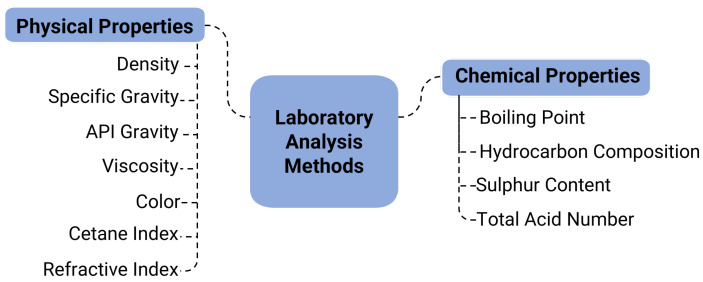
Lab analysis classification of physical and chemical properties.

Lab analysis consists of various physical and chemical tests, accompanied by a detailed explanation of product properties (Figure 5). Physical tests typically include analysis of density, API gravity, viscosity, water and sediment content, pour point, flash point, and colour. Meanwhile, chemical analysis comprises assessment of hydrocarbon composition, boiling point, acid number, sulphur content, carbon, oxygen, and metal content [65]. The results of the laboratory analysis reveal the crude oil product’s actual properties and provide an accurate prediction of its yield. However, laboratory analysis is a time-consuming process and has a high cost due to the test equipment and personnel required, which constrains the analysis. Typically, lab testing is conducted within 48-72 h to obtain a detailed assessment.

### 4.1. Gas Chromatography

In practice, yield estimation can be performed by using gas chromatography (GC) to analyse the hydrocarbon composition and boiling point. GC is an analytical technique used to separate and analyse the chemical components present in a mixture. GC requires a detector to separate and characterize the crude oil, such as mass spectrometry (MS), a flame ionization detector (FID), a time of flight mass spectrometer (TOFMS), or a flame photometric detector (FPD) [66,67,68,69,70,71,72,73,74,75,76,77,78,79]. The chromatogram graph generated from GC shows different peak sizes, where the number of peaks indicates the compounds present, and the size of each peak represents the amount of each compound. The boiling point and hydrocarbon composition obtained from the graph can be used to calculate the yield percentage by integrating the area under each peak. From the mass or volume percentage, the true boiling point (TBP) curves can be plotted, and the yield can be calculated directly based on the cut point ranges of each crude oil fraction. The TBP is the most crucial characteristic of petroleum as it is helpful for identifying the product yield, selecting the correct fuel fraction, designing the operating units, and understanding the oil’s behaviour before carrying out the distillation process [80,81].

Some studies have been conducted on the usage of GC. In [82], a comparison was performed between physical distillation using ASTM D-2892 and ASTM D-5236 [83,84], and simulated distillation (SIMDIS) using GC. The study aimed to improve the measurement technique for assay distillation and reduce the uncertainties of yield estimation. The analysis was performed to detect a mixture’s composition in the range of C_5_ to C_44_. The results indicated a high level of engagement between the two methods; however, SIMDIS yielded a higher confidence level with a lower deviation error. Its detection was faster and more reliable, based on a reduction in analysis time from 48 h using physical distillation to 4 h using GC. As it is a temperature-dependent process, GC is successful in identifying light-to-medium hydrocarbon compositions; however, it exhibits complications when used on mixtures with higher boiling points due to their lower volatility. A summary of physical distillation and SIMDIS is tabulated in Table 1 for a better overview.

Another study explains the setup of GC for characterizing crude oil without any preliminary separation of light and heavy fractions [85]. This new methodology was introduced to address the needs of the pretreatment process prior to GC analysis, including the use of distillation, backflush, purge, and trap techniques. A thermal extraction device is attached to the GC, which is functionalized to extract light components from the adsorbent matrix using thermal energy and transfer them to the GC unit. Meanwhile, heavy fractions of the sample remain in the adsorbent material. The adsorbent material can be varied, as it differentiates the ranges of hydrocarbons in the analysis.

A study by [86] compiled the results of crude oil characterization using PIONA, GC-FIMS, and SIMDIS. The PIONA test is used for the detection of paraffin, isoparaffin, olefin, naphthenes, and aromatics, and is commonly used for characterizing crude oil below 200 °C. In contrast, GC is used for the middle fraction, with a boiling temperature of 200–360 °C. The chromatogram of each hydrocarbon can be converted to a boiling point distribution scale using retention time calibration. A good agreement was recorded between the combined PIONA and GC-FIMS result and the SIMDIS curve. The integration of FIMS-SIMDIS provides in-line data consistency with stand-alone SIMDIS, except for at boiling points above 250 °C, where it is found to have a higher mass fraction than the SIMDIS data curves. Mass spectrometry (MS) and a flame ionization detector (FID) were integrated for gas characterization. The results showed comparable and similar outcomes for both methods, indicating the successful development of the reconciled method using PIONA and GC-FIMS.

There is also research on the use of two-dimensional GC coupled with time-of-flight mass spectrometry (GC × GC-TOFMS) for the characterization of PIONA in kerosene (PIONA GC) [87]. As mentioned previously, PIONA characterization was conducted for a petroleum mixture with a boiling point below 200 °C. Since kerosene is located in the middle distillates, GC × GC was introduced for better resolution power, high peak capacity, and sensitivity. The results revealed that combining both FID and TOFMS provides a detailed, semiquantitative analysis of kerosene, especially in terms of saturated and aromatic contents for PIONA analysis. The study emphasizes a comparison of GC × GC-TOFMS and PIONA through semiquantitative analysis. For the saturated component, the difference ranges from 0.35 to 3.30%, while the aromatic component is in the range of 3.3% to 45.9%. It is also mentioned that n-paraffin distribution, typically obtained by GC-FID, enables a rapid overview of the boiling range distribution and characterizes the fraction from a mixture of multiple cuts. The simple and convenient use of GC has largely eliminated the need for most physical tests and analyses.

### 4.2. Physical and Instrumentation Analysis

Although GC provides details on the estimation of petroleum product yield, characterizing some physical properties remains crucial. Density, specific gravity, and API gravity are essential analyses for understanding the properties of crude oil. An increase in the density value directly increases the content of aromatic compounds; however, an increase in saturated compounds causes a lower density value [15]. According to the previous analysis, the density of crude oil ranges from 0.8 to greater than 1, corresponding to lighter to heavier crude oils. Meanwhile, for API gravity, the trend interpretation is inverse to that for density; a higher API value indicates lighter crude oil, and a lower API value indicates heavier crude oil.

The measurement of density and specific gravity is generally conducted using a hydrometer, pycnometer, and digital density meter. Each of these instruments has its own measuring standard, following the ASTM [15,88]. The suitability of the detection instrument depends on the sample size and practicability. A density meter is a fast and reliable instrument, and is widely applied for measuring light-to-medium crude oil. For heavy oil and bitumen, the surface tension of the sample affects the measurement, suggesting that only a hydrometer is suitable. Meanwhile, a pycnometer is typically used for small sample sizes. Another important analysis is the viscosity of crude oil, which explains the liquid’s capability to flow. At a lower viscosity, the liquid flows more easily. Conceptually, this depends on the content of polar molecules, such as resins and asphaltenes [89]. A higher viscosity indicates a high yield of heavy distillate and a lower percentage of light distillates, besides the generation of water or oil emulsion [90]. For the viscosity characterization, capillary types, an orifice, or a viscometer can be used to record the viscosity of crude oils with a fast and reliable technique [15].

Sulphur content also exhibits a similar trend, with a higher sulphur content in the crude oil composition resulting in a higher proportion of heavy distillates. For conventional crude oil, the sulphur content varies from 0.1% to 3% *w*/*w*; meanwhile, heavy oil and bitumen have a higher percentage in the range of 5% to 6% *w*/*w* [15]. Temperature also impacts the sulphur content by causing the migration of sulphur to heavier components at higher temperatures and simultaneously reducing its content in liquid products [91]. Measurement of the sulphur content in gases, liquids, and solids can be performed using a sulphur analyser or X-ray fluorescence (XRF) analysis, which may require some sample preparation steps. Additionally, the total acid number is another important analysis as it indicates the acid composition in crude oil. The acid number is classified based on the acid content per milligram of potassium hydroxide (KOH), and is commonly in the range of 0.05–6.0 mg KOH per gram of sample [15]. The test method typically involves potentiometric titration or colour indicator titration by dissolving the oil sample and titrating with KOH. TAN does not entirely reflect the corrosiveness of crude oil; however, a high TAN increases the potential for affecting refinery unit operations. Water and sediment analysis is also crucial due to their potential impact on feed volume, as well as their influence on operational conditions and pipeline safety.

### 4.3. Spectroscopy

Spectroscopy is one of the analytical techniques used to determine the chemical properties of crude oil, including functional groups and molecular structure. The application of spectroscopy provides an alternative analysis to enhance traditional assay characterization, which is often hindered by tedious lab work, time consumption, and cost inefficiency [92,93]. The measurement and identification of chemical structures is performed using the adsorption and emission of radiation or light that can penetrate the sample. In general, spectroscopy is categorized into several techniques, for example, Raman spectroscopy, Infrared (IR) spectroscopy, Ultraviolet-Visible (UV-Vis) spectroscopy, Nuclear Magnetic Resonance (NMR) spectroscopy, and X-Ray spectroscopy.

As reported by [94], Raman spectroscopy has minimal interference, is safe, offers a high resolution, does not require any sample preparation, has a low operation time, and has excellent replicability. Meanwhile, for IR spectroscopy, variation in infrared absorption by molecules affects the vibration and rotation of energy levels. This technique enables fast response analysis through chemometrics and statistical data analysis, has a low cost, and provides accurate data replication [93]. NMR spectroscopy provides a comprehensive analysis of an oil’s physical and chemical properties through its structural information [95]. The properties of crude oil greatly depend on the type and number of hydrocarbons, such as aliphatic, aromatic, and naphthenic components, as well as other components with high molecular weights. Fluorescent spectroscopy also exhibits good potential for the analysis of crude oil based on its good optical detection, high sensitivity, simple instrumentation, and suitability for portable analysis [96,97].

Most spectroscopy techniques are combined with mathematical modelling and machine learning for the prediction of yield. Manual interpretation of spectral data requires extensive knowledge and time for analysis, which drives the development of automation and AI applications [92]. In most cases, spectroscopy techniques provide data in the form of spectra, and then an AI algorithm transforms this data into a yield prediction. One study utilized attenuated total reflection IR spectra (ATR/IR) and simple PLS regression to predict crude blend yields [98]. The development of the PLS model involved a combination of pretreatment methods and wavenumber range selection, which was achieved by integrating several different regions. The findings demonstrated good accuracy between the crude assay and simulated data for pure crude oil. However, the predicted value showed reduced prediction accuracy for non-pure crude oil. For example, inclusion of non-crude oil in the blend caused a difference close to 15%, especially for the high-boiling-point fraction. Another study also employed PLS regression with NMR spectral data to evaluate crude quality and integrated the UOP characterization factor K for crude property classification [99]. The PLS model enabled good prediction of the Kuop factor, total acid number, and TBP distillation yield. Meanwhile, less accurate predictions for density and sulphur content demonstrated data dispersion between measured and predicted values. Data preprocessing and pretreatment are compulsory in order to reduce the noise region and simultaneously improve prediction.

NMR is also able to predict the saturates, aromatics, resins, and asphaltenes (SARA) fraction of crude oil by developing SARA correlation using the aromatic factor and MATLAB software [100]. The aromatic factor of hydrogen and carbon was calculated using Dickinson’s equation. This study yielded good prediction and high accuracy, with an error range of 0.1–9.4%, and concluded that the aromaticity factors increase with increasing crude oil gravity. Moreover, the application of data-driven methods has successfully predicted crude oil yield using data from FTIR spectra [92]. This study employed two methods for reducing the dimensionality of FTIR spectra: principal component analysis (PCA) and an autoencoder, in conjunction with support vector regression (SVR). The prediction using autoencoder/SVR yielded slightly higher accuracy compared to PCA/SVR; however, the PCA/SVR model demonstrated greater simplicity due to its linear PCA component.

Another study utilized NIR spectroscopy and a convolutional neural network (CNN) to analyse the composition and proportion of simulated blended crude oil [101]. Spectral information was extracted by applying first-order derivative processing, wavelength selection, and PCA before being introduced to the CNN model. The FTIR spectrum required preprocessing due to significant data overlapping, hidden features, and noise cancellation. The CNN model demonstrated high feasibility and accuracy for crude oil estimation, with an R-squared of more than 0.98. It was concluded that the spectroscopy method allows for fast prediction and reduces the need for laboratory work and comprehensive sample preparation, while providing high-value information.

## 5. Development of Process Simulation

Process simulation plays a crucial role in the oil refining industry, as well as in yield estimation, by simulating the operation of refinery units. It offers an excellent methodology for optimizing production efficiency, predicting product quality, and monitoring operations. The simulation takes a real-world application and integrates it into a system, adapting it to suit the software’s capabilities. Refiners are authorized to develop and optimize various operations and processing conditions, analyse the behaviour of crude oil, and predict product fractions. Advanced computational tools provided by process simulation enable better decision-making, maximize high-value products, and control the operation’s sustainability in terms of both economics and the environment. Process simulation has been employed in oil refineries since the 1960s to design complex industrial production processes, encompassing all chemical processes and oil refining operations. Over 60 years, various simulation process softwares have been developed and are being widely applied worldwide. The process simulation is embedded with fast response calculations and complex iterative algorithms, which are highly suitable for the integrated process.

In general, oil characterization involves specifying the properties of crude oil prior to simulation, either by using existing assay data or creating new data. Boiling points, such as the TBP or any ASTM standard, and density or molecular weight are the compulsory input properties for the software to develop pseudo-components within crude oil. The inclusion of a light hydrocarbon composition in oil characterization enhances simulation prediction as it supports the initial approximation. The simulation also requires configuring fluid packages for the thermodynamic model to calculate the vapour–liquid equilibrium. There are several types of fluid packages available, including the Peng–Robinson (PR), Soave–Redlich–Kwong (SRK), Non-Random Two Liquid (NRTL), and UNIQUAC models, among others. Most process simulations for crude oil and hydrocarbons apply the Peng–Robinson (PR) thermodynamic model due to its robust performance and high suitability. The PR model provides a correct prediction of properties around the critical point, both temperature- and pressure-dependent, and the state of the equation follows the properties of hydrocarbon mixtures [102]. The PR model is suggested to enhance hydrocarbon simulation based on it being an improved version of the SRK model, providing a well-balanced approach in terms of combining precision with a high level of simplicity.

This section focuses on the available process simulation tools in the oil refining industry, specifically for process optimization and yield prediction, including AspenTech, Petro-SIM, and UniSim design simulator. A summary of different simulation softwares is tabulated in Table 2 for a better overview of this section.

### 5.1. AspenTech

**Figure 6:**
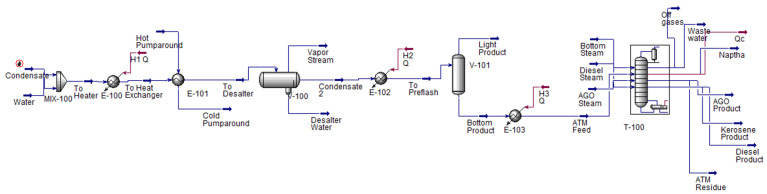
Process flow diagram for crude oil condensate distillation unit simulated in Aspen Hysys software (V14), in which blue arrow: mass stream and red arrow: energy stream.

AspenTech has been innovating since the 1980s under the Project Advanced System for Process Engineering (ASPEN) [110]. AspenTech serves as a platform to help customers globally achieve their operational and sustainability goals for process design by leveraging its simulation background. Aspen simulation is not limited to process simulation, but also encompasses economic and environmental analysis, dynamic simulation, and AI integration. A comprehensive prediction of the liquid–vapour equilibrium, heat and material balances, and chemical engineering equipment is implemented, utilizing over 30 thermodynamic models that focus on fluid property computation [111]. The chemical, petrochemical, energy, and oil and gas industries have widely applied the software. Practical usage of process simulation involves specifying input properties and thermodynamic models, building the process flow diagram, running the simulation, and analysing the simulated results. In the industrial process of oil refining, the distillation column is the primary operation unit, where products are separated based on their boiling points. Meanwhile, the addition of other operations units is not specified, as this depends on the process target and crude oil feed conditions. An example of process simulation is presented in Figure 6.

#### Case Study

A study by [112] on the process simulation of different grades of Basrah crude oil was successfully carried out using a desalter, separator, heater, and three-sided stripper distillation column. The distillation column was equipped with 29 trays. The feed entered at tray 27, and product stripping was performed at trays 11, 17, and 23 for kerosene, diesel, and atmospheric gas oil (AGO), respectively. The column operating conditions were specified at individual fraction cut points, with 95% recovery at temperatures of 289.5 °C (kerosene), 365.4 °C (diesel), and 507.1 °C (AGO). Thus, the production rates of distillates showed a distribution of 11.54%, 30.70%, 19.55%, and 2.33% for naphtha, kerosene, diesel and AGO, respectively. The simulation could successfully produce 2 × 10^4^ barrels per day, aligned with the study’s intention to achieve 100 million barrels per day. The study also focused on the use of simulation for economic analysis, which resulted in a positive net present value (NPV); however, further sensitivity analysis and consideration are needed for a comprehensive assessment.

Another study on yield estimation using Sarir crude oil demonstrated good agreement between simulation and lab results [113]. The simulation followed ASTM D86 [114] curves, with the cut point temperatures of the end products specified as 90 °C, 160 °C, 221 °C, 327 °C, and <550 °C for light naphtha, heavy naphtha, kerosene, diesel, and residue, respectively. A steady-state model of crude oil refining comprised a heater, desalter, distillation column, and depentanizer for naphtha separation. The distillation column consisted of 34 trays, with a feed stream added to the column at tray 31, and two side strippers for kerosene and diesel products, withdrawn at trays 12 and 22. It is observed that both studies located the feed tray within 2–3 trays from the bottom of the distillation column, as the bottom part of the column induces a higher temperature for efficient separation. Process simulation provided a higher product flow rate compared to the refinery yield, with a maximum error of 12.5% for kerosene. The study also found that the addition of preflash before the distillation column does not improve product quality, but it reduces the energy consumption of the furnace and distillation process.

Additionally, Ref. [115] conducted a study on the effect of parameters such as cut temperature, top tower temperature, crude feed temperature, steam flow rate, and tower pressure on the yield of kerosene using Aspen Process Simulation. It is stated that variations in process conditions resulted in fluctuations in product yield. To increase the yield of light products, such as naphtha and kerosene, reducing the top pressure of the distillation column is the best option, as it incurs no additional operating costs. An increase in the steam stripping flow rate and a decrease in the cap temperature can also improve the flow rate of light distillates. However, increasing the feed temperature is not highly recommended due to the high energy consumption and cost of heating the crude oil. Each of the process adjustments influences the product yield, yet the selection of optimal processing conditions depends on the refinery’s preference and economic considerations.

The involvement of process optimization simultaneously improves modelling, and this is explained by a study that used Aspen HYSIS integrated with in-built Sequential Quadratic Programming (SQP) [116]. SQP was implemented to optimize the mole fraction, with the total production rate set to its maximum limit using the spreadsheet function in the software. At the early stage of simulation, a comparison of the mole fraction and the stripping tray between the HYSIS model and plant data yielded a maximum deviation of 3.2% and 23.2%. Thus, the optimization improved the mass flow rate of the end products by a maximum increment of 1.9% for naphtha. The mole fraction and tray withdrawal temperature also increased. The study concluded that the temperature, pressure, mole fraction, and molecular weight increase as the tray number increases and as one goes down the column.

A transition from a steady-state model to a dynamic model improves accuracy, optimizes plant design, and refines control management in the system [117]. With the application of Aspen software, a well-constructed steady-state model can be easily converted into a dynamic model. The modelling includes crude oil characterization, true boiling point (TBP), and various unit operations, such as the preflash, heater, and distillation column with three side strippers. The transition steps involve specifying pressure and flow relationships, boundary streams, equipment sizing, and control system strategies. The volume of the condenser and reboiler, as well as the geometry of the column tray, are compulsory for the distillation column, which requires specification prior to dynamic shifting. Additionally, a proper control strategy is a key factor in dynamic modelling for disturbance reduction, as even a minor variation can cause significant disruption to the entire system.

The design of unit operations and the distillation column in AspenTech has a significant influence on the end products. The specification of the product’s cut point temperature typically depends on the refiner’s preferences and the target end product, but must fall within the product’s boiling temperature range. This is necessary to prevent a mixture of products and to control product quality. A wider cut point temperature range for a specific product simultaneously increases its mass flow rate and reduces the flow rate of other products. Additionally, the withdrawal tray stages also determine the final product quantity, following the temperature difference across the crude distillation column.

### 5.2. Petro-SIM

**Figure 7:**
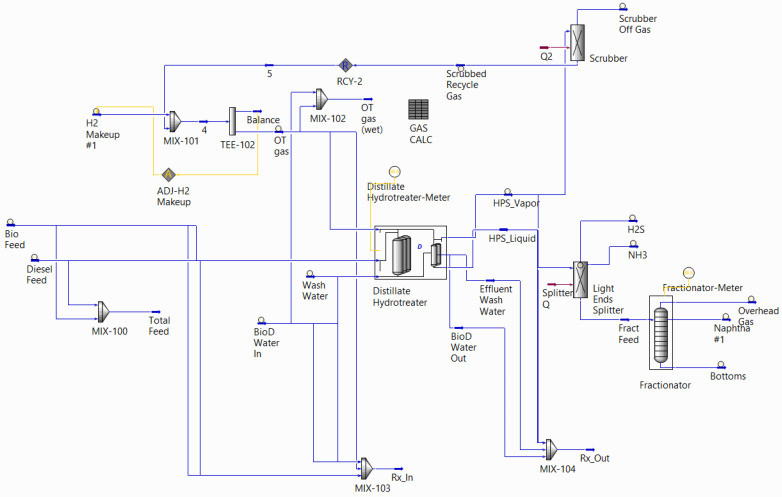
Process flow diagram for renewable diesel reactor setup simulated in Petro-SIM software, in which blue arrow: material stream, red arrow: energy stream and yellow arrow: adjust stream. Adapted from [118].

Petro-SIM simulation software, developed by KBC Advanced Technologies in 1979, focuses on process simulation in the petrochemical and oil and gas refining industries. The software prioritizes unit operation modelling through assay specification by regulating process conditions and establishing good flexibility with crude oil properties. Petro-SIM also applies mass and heat balance concepts in simulation to accurately predict energy generation and consumption [107]. This software enables comprehensive performance management and control by providing an action list for optimization, such as tray and equipment specifications. The unit operations available and the interface in this simulation have some similarities with those for AspenTech discussed in the previous section in Figure 7, as it uses the same code; however, it has limitations in dynamic simulation and is more concerned with the real-time application.

#### Case Study

A study on process simulation for PETRONOR Refinery, Madrid, demonstrates the successful inclusion of the following units: atmospheric and vacuum distillation columns, fluid catalytic cracking (FCC), visbreaker, mild hydrocracker, and catalytic reformer [119]. The unit specification begins with a rigorous unit simulation, followed by a non-rigorous unit, and a spreadsheet unit is utilized for property alteration and yield calculation. Petro-SIM enhances refinery processing plans by building a prediction model for calibration and unit monitoring, thereby simultaneously improving refinery optimization. Manipulation of key process variables, such as the crude mixture, TBP cut point, routing streams, and product blending, optimizes the model and product quality. The simulation should generate a high level of data accuracy, and this is achievable by keeping the unit operation updated and following the real process in a refinery. In addition, the study successfully develops a comprehensive refinery process, including optimization, which involves multiple specialized parties. Good communication between all parties involved is key to success.

Another study by [120] investigated maximizing oil recovery in a gas–oil separation plant (GOSP) by developing a Petro-SIM process simulation. The initial pressure, volume, and temperature (PVT) of each unit operation acted as the feed information for the simulation. The oil input properties also included the composition of the feed stream and the hydrocarbon stream. A process steady-state model was developed with high-pressure and low-pressure separators (HPS & LPS) and a stabilizer, each incorporated with a stage and equipment size. This simulation model was connected to a spreadsheet for data collection and a built-in optimizer for optimal processing conditions. The study concluded that the sizing of equipment does not impact oil recovery; however, pressure and temperature cause variations in the end product due to the complex, multicomponent nature of hydrocarbons. Small fluctuations in temperature and pressure directly altered the composition and phase distribution of the oil separation.

Petro-SIM software was applied during continuous catalytic reforming (Octanizer), and was able to predict the production of gasoline [121]. The addition of process specifications and operational data for the feed stream and column, including temperature, purity, catalyst density, and flow rate, was performed in the early simulation. As the Octanizer involves a kinetic reaction, catalyst density provides sufficient information for performance evaluation. It is noticeable that the calibration process was conducted in the REF-SIM module embedded in an Excel spreadsheet before being transferred to the Petro-SIM software. The process simulation provides a promising approach to addressing changes in processing conditions, identifying problems, and controlling operational costs. A comparison of gasoline products between the simulation and actual data yielded similar results, but with different temperature cut point percentages. Notably, the temperature of the product percentage cut for the simulation was lower than the actual temperature, especially for percentages <10% and >70%.

Although Petro-SIM and AspenTech share some similarities in process simulation, the focus of application differs between these two software programs. AspenTech primarily focuses on comprehensive chemical process simulation, encompassing various unit operations and thermodynamic models. However, Petro-SIM serves as a platform focusing on upstream and midstream hydrocarbon simulation for the oil and gas industry.

### 5.3. UniSim Design Simulator

UniSim simulation software is a package developed by Honeywell that simulates complex processes and enables the development of automatic control systems for the oil, gas, and petrochemical industries. Roughly, UniSim simulation development follows similar steps to those of Aspen; however, more focus is placed on operational training, control, and dynamic simulation with automated calculations [108]. As mentioned previously, crude oil is specified, followed by the transformation of the feed chemical component and physical properties into pseudo-components, which is simulated in the software database [102]. The alteration is completed with information provided by the boiling temperature and distillation curve. The specification of the thermodynamic model is compulsory before developing unit operations based on the feed composition and end product.

The literature and case studies on process simulation using UniSim software are limited and this has not been widely explored. One study utilizes an absorption column equipped with a reflux drum and condenser, and the column is specified to have 21 stages, with the feed tray located at stage 17 [122]. The side stripper for kerosene and diesel is also added at stages 7 and 13, respectively. The study states that the temperature of gasoline, kerosene, and diesel increases as the product flow rate decreases. A comparative study on UniSim design and Edmister–Okamoto (EO) correlation for the atmospheric distillation column was conducted [123]. The development highlighted significant deviation in the diesel fuel flow rate. The EO method only provides a rough estimation of the distillation column with high substantial error, particularly with heavy hydrocarbon components. Meanwhile, for light products, the error is approximately below 11%, with the lowest error being 0.62% for kerosene.

Another study was conducted on the steady-state process of a crude oil distillation column by varying the heat exchanger arrangement, simulated using UniSim software [124]. Conversion of assay data into hypo-component data provided property plots, composition curves, and product distribution plots for an initial overview of the crude oil. This study employed optimization to minimize the overall heat transfer coefficient (UA) for an optimal split ratio. A plant case study with a prefractionating column provided a good model for simulation development and yielded the lowest UA value, explaining the importance of unit operation selection and arrangement. It was concluded that the arrangement of heat exchangers affects the molar flow rate and heat flow in the process design.

Process simulation provides fast, reliable, and cost-efficient yield prediction for petroleum products. The simulation interface enables easy understanding and control to facilitate optimization and changes. Several key parameters influence the simulation results for the distillation column, including temperature and pressure, stage number, cut point temperature, and reflux ratio. The application of process simulation can significantly reduce costs and time compared to laboratory analysis; however, a proper simulation is required to ensure the consistency and accuracy of predictions.

### 5.4. Alternative Simulation Software

In addition to the simulation software discussed earlier, various other tools are available for process simulation, each offering varied functionality. ChemCAD software has been used for simulating the reactive distillation process of acetic acid and ethanol [125]. This software requires identifying unit operations prior to designing feed properties, selecting a thermodynamic model, and specifying operational information. The simulation can provide consistent results, with experimental results showing an 4.9% increase in ethyl acetate, the top product of the reactive distillation. It also provides some graphical data to facilitate a better understanding of the chemical reaction occurring in the column. Furthermore, ChemCAD software has also been applied to simulate the production of biodiesel (methanol) under different scenarios, such as as a raw material and electricity source, for life cycle assessment (LCA) [126]. The simulation successfully modelled the methanol product in various scenarios, yielding comparable findings. Another successful simulation was carried out by utilizing VMGSim to simulate the mono-chlorobenzene plant unit [127]. VMGSim provides 2000 samples of simulation data for model algorithm development, including variants in both nominal and faulty operation.

In a study by [128], simulation of process design in offshore petroleum production was conducted using open-source DWSIM software and compared to simulation with the widely used software Aspen Plus. Both software applications apply the Peng Robinson thermodynamic model; however, in Aspen Plus, the specification of feed composition is essential at the early stage of simulation. The study concluded that both simulations provided a strong alignment with the real plant data, and there was only a <5% difference between the simulations. Additionally, the simulation software OpenModelica integrates ChemSep and DWSIM for the property database and thermodynamic algorithms, respectively [129]. ChemSep is a systematic and open-source database with availability as a column simulator for process simulation [130]. The study found that OpenModelica exhibits excellent alignment with simulated results from DWSIM and ChemSep in various thermodynamic model cases.

The application of process simulation has been extended to a broader scope, including particulate materials and the agricultural sector. Dyssol is an open-source software used for designing processes involving particulate materials by integrating a sequential–modular approach [131]. This approach ensures the complete description of a single process step using a mathematical model and provides solid-phase specification in various dimensions of interdependent parameters. Dyssol simulation provides a flexible, customized, and comprehensive system for processes involving granular materials. Furthermore, the COCO simulator has been used to model a gasification unit of agricultural residue [132]. The COCO simulator, combined with MATLAB operations, provides a consistent and steady reaction for the gasification of sunflower husk and apple trees. In this simulation, various terms were used, including TEA for the thermodynamic system, CORN for the reaction package, and COFE for the CAPE-OPEN flowsheet engine. The comparison of simulated and experimental results demonstrated model validity and reasonable agreement.

## 6. Process Modelling and Machine Learning

Advancements in process operations in the oil and gas industry have enabled the successful integration of process modelling and machine learning into various applications, particularly in process control, prediction, and optimization. As discussed in the previous section, laboratory analysis and process simulation can effectively estimate the yield of crude oil; however, some limitations exist in the execution of modelling and machine learning. The newest technology allows for fast prediction, high accuracy, and reliable methodology without the need for expensive equipment, chemicals, and software. Moreover, modelling implements actual phenomenon and time series data, which are closely related to the current operational status.

Mathematical approaches enable the observation, interpretation, and prediction of actual situations. Machine learning is a subdiscipline of artificial intelligence (AI) that highlights pattern development based on historical data for estimation and optimization. The model opts to perform automatic learning using provided data. The number of data points defines the precision and accuracy of the model prediction. A comparison between mathematical modelling and machine learning is presented in Table 3 for a clearer overview.

### 6.1. Mathematical Modelling

Few case studies have been conducted on the mathematical modelling applications used to predict product yield in the oil refining industry. In a study by [133], a simple supervised mathematical model was applied to accurately estimate the mass fractions of naphtha, kerosene, and diesel within a temperature range of 70–150 °C, achieving absolute errors of 12% *w*/*w* using the conventional system. From the full dataset of TBP curves generated by their distillation system, six data points from each sample (S1–S6) were selected to train the mathematical model. These inputs included three temperature values (*T*2): the lowest (*T*2_1_), medium (*T*2_2_), and highest (*T*2_3_), along with their corresponding accumulated masses (*M*_1_, *M*_2_, and *M*_3_). The relative concentrations (*C*r) of the oil derivatives were modelled using coefficients *c*_*i*_ as follows in Equation (1):

(1) $$C_{r}=c_{1}\left(\frac{M_{1}+M_{3}}{M_{2}\times T2_{1}}\right)+c_{2}\left(\frac{M_{1}}{M_{2}}-\frac{T2_{3}}{T2_{1}}\right)+c_{3}$$

In another study, a first-order mathematical model, based on Volk’s model for the delayed coking unit, provided a precise approximation of the actual yield data [134]. The delayed coking unit is a conversion process focused on the separation of gas, liquid and solid coke. The gas product consists of C_1_–C_5_ light hydrocarbons, hydrogen, and hydrogen sulphide, while the liquid product comprises gasoline and gas oil. The improved Volk’s model involves five key parameters in prediction, including temperature (*T*), pressure (*P*), liquid space velocity (*LSV*), and feed microcarbon residue (*MCR*) content. The study proposed a modified Volk’s model for each product yield, as presented in Equations (2)–(5). The calculated yield estimation exhibited a very close trend to the commercial data compared to other predictive models, with an average error in the range of 0.25–15%.

(2) Gasyield=0.111MCR−0.012T+0.088P−507.499LSV+4.364,

(3) Gasolineyield=0.174MCR−0.007T+0.088P−462.43LSV+9.287,

(4) Gasoilyield=0.390MCR−0.045T+0.337P−1535.109LSV+20.986,

(5) Cokeyield=1.084MCR−0.070T+0.1762P−368.649LSV+75.024,

A study by [135] simplified the six-lump model by considering mass balance, a kinetics equation, the Nelder–Mead minimization algorithm, and the Runge–Kutta method for yield estimation in a hydrocracking reactor. A six-lump model that correlates crude oil and products (diesel, kerosene, heavy naphtha, light naphtha, gas) was developed under the assumptions of a hydrocracking process co-occurring, a heavier lump transforming into a light lump in between two lumps, an irreversible first-order reaction, and an adiabatic process with no back-mixing and coke formation. Then, the model kinetic parameters estimated by the Nelder–Mead minimization algorithm were used for simulating both the static and dynamic models. The findings demonstrated significant prediction accuracy, with a mean deviation of 0.0036 and 0.0021 for the static and dynamic models, respectively, between plant data and simulated results for kerosene.

Partial least squares (PLS) methods are widely used in process industries for handling data correlations in soft sensor modelling. The initial step in PLS involves identifying principal components (PCs) from high-dimensional process variables (X and Y), followed by establishing relationships between the resulting latent variables. However, conventional PLS has certain limitations: (1) it requires large datasets for effective generalization, and (2) it operates based on a linear framework. This is because nonlinear variants, such as neural network PLS (NNPLS) and kernel PLS (KPLS), face challenges in selecting appropriate nonlinear parameters. For example, in a case study, a PLS-based model was compared with a deep neural network (DNN)-based soft sensor for predicting the cut point temperature of heavy diesel, which also affects product yield and quality [136]. Process variables, including temperatures, pressures, and material flows, were collected through the plant’s Distributed Control System (DCS). The DNN model’s predictions showed significantly better alignment with actual values compared to the PLS model. This outcome highlights deep learning’s capability to effectively capture nonlinear latent features, making it a highly suitable and promising approach for soft sensor modelling.

Prediction of petroleum products from crude oil was successfully achieved using the mathematical model. For example, the developed mathematical model, such as Volk’s first-order model for delayed coking, can predict the yield of gas, gasoline, gas oil, and coke. Another equation, the six-lump model, involves mass balance, a kinetics equation, Nelder–Mead minimization, and Runge–Kutta, and can yield high-accuracy predictions in both static and dynamic modes. Applying this mathematical model technique enables precise estimation, reduces resource usage, and helps illustrate and define the system.

### 6.2. Machine Learning

The widespread application of machine learning in the petroleum industry for process control has led to impactful findings. Machine learning (ML) has been increasingly applied in the petroleum industry to enhance the accuracy and efficiency of estimating petroleum product yields. ML techniques such as Random Forest (RF), Artificial Neural Networks (ANN), and Gradient Boosting Regressor (GB) have been effectively used for the prediction process. These models utilize historical production data and real-time information, along with various input parameters, to deliver accurate forecasts, even in complex scenarios involving multiple shut-ins. The development of a machine learning-based prediction model for a distillation process involves data collection, characteristic extraction, normalization, and algorithm selection [137]. The prediction of product yield and specification thresholds for end product quality by ML models is successfully presented in the existing literature, as summarized in Table 4.

The ANN model applies a mathematical algorithm, artificial neurons, that mimics human brain functions to predict the interaction between input and output. It is compatible with a wide range of input features and provides precise estimation of near-infrared spectroscopy data, offering detailed molecular insights. The support vector machine (SVM) model focuses on classification and regression, employing a kernel function to conduct data linearization at a high performance rate and with high simplicity, as well as exhibiting advantages in handling small datasets. The Gaussian Process Regression (GPR) model is a multidimensional, non-parametric, and nonlinear tool that follows Bayesian probability theory to assess scattered datasets.

#### 6.2.1. Neural Network

ANNs have been utilized to develop nonlinear models for estimating product yield in distillation columns. The development of an ANN-based prediction model for a distillation process involves collecting data, extracting characteristics, normalizing the data, and selecting an appropriate algorithm. The developed model can predict the desired output directly or indirectly using multivariable inputs. For instance, one study utilized a nonlinear autoregressive with exogenous input (NARX) structure, a type of ANN algorithm, to directly predict the mole fractions of distillate and bottom products based on input variables like heat duty and the reflux flow rate. The model demonstrated high reliability and low operational costs [138]. Based on a similar concept of an ANN-based estimator, the composition of the distillate was predicted. The ANN model has forward-flowing information in predictive mode and back-propagated error corrections in learning mode [139]. In this study, the input vector consisted of 17 temperature entries of 15 trays, a reboiler, and a reflux drum. Meanwhile, the output vector of the estimators consisted of five liquid and five vapour distillate compositions. The results revealed comparable findings to the developed simulation program, and the ANN model saved 68.75% time without compromising accuracy [139].

Another study by [140] developed a soft sensor model for predicting light naphtha vapour pressure (RVP) in the crude distillation unit. The soft sensor encompasses multiple linear regression (MLR) analysis and neural network models, including linear neural networks (LNN), multilayer perceptrons (MLP), and radial basis function (RBF) networks. The findings indicated that MLR analysis is susceptible to deviations from the experimental values. By using a neural network, the LNN model demonstrated lower deviation and better results than MLR for both oil types. Meanwhile, the application of MLP and RBF models yielded a higher correlation coefficient, based on a good sensitivity ratio, and they were shown to be acceptable for process monitoring and prediction. The study also concluded that naphtha RVP is influenced by the conditions at the splitter top, such as the temperature of the domestic oil and the pressure of the reboiler oil.

**Table 4:** Application of ML models in oil and gas industries.

Model Types	Objective	Unit Process Applied	Advantages	Limitation	Ref.
ANN	Gasoline and butane concentration	Debutanizer	Able to overcome delay. Enables efficient, low-cost, real- time estimation.	Prediction depends on data variables’ quality. Requires data preprocessing.	[141]
ANN	Distillate composition	Distillation column	Handle many inputs with accurate results.	Manual tuning of synaptic weight and threshold reduces classification error. Uncertainty in controlling product composition.	[139]
ANN	Mole fraction of distillate product	Binary distillation column	Satisfactory estimation performance. Enhances overall control. Enables fast response.	Selection of secondary variables (nature and location).	[142]
ANN	Product composition	Reactive distillation column	Allows error refinement. Capability to manage composition under dynamic settings.	Complex unit operations delay model development.	[143]
ANN	Top and bottom composition, reflux ratio	Batch distillation	Sped-up training improves prediction.	Choices of suitable model optimization.	[144]
RANN	Product composition	Batch distillation	Good agreement with actual values.	Consistency of model prediction under normal and noise temperature.	[145]
Adaptive NN	Product composition	Binary distillation column	High accuracy with faster response.	Low efficiency with high input and multicomponent mixtures.	[146]
XGBoost	Ethane and ethylene composition	Batch distillation	Only requires temperature and pressure sensors.	Requires intense data preparation.	[147]
SVM-Bayesian	Product yields	Hydrodesulfurization process	Can handle nonlinear complex data.	Multiple factors affecting SO_2_ removal efficiency.	[148]
SVR-GA	Product yields	Hydrodesulfurization process	Improves accuracy and alignment with expected values.	Requires dataset fine-tuning.	[149]

In the indirect neural network method, the model predicts temperature, which is crucial for controlling the crude distillation column and can be used to estimate the steam consumption required to maintain the production stage temperature, which impacts the distillate yield [137]. The model’s components include the choice of algorithm, optimization method, and batch size, which significantly influence the model’s accuracy. This study reveals that the use of ANN algorithms, such as long short-term memory (LSTM), is effective in predicting production stage temperature with a high coefficient of determination and a low root mean squared error [137]. ANN is one of the most frequently applied machine learning methods due to advancements in the implementation of human-like computational strategies for output prediction. This algorithm also offers a fast processing time of a few minutes and provides accurate predictions with high sensitivity.

#### 6.2.2. Support Vector Machine

SVM models have been increasingly adopted in refinery operations for predictive analytics due to their ability to model nonlinear relationships and effectively handle high-dimensional data. One notable application is in the prediction of crude oil properties, such as saturation pressure, a crucial parameter that influences product quality. Traditional empirical or linear regression models often fall short in capturing such nonlinearities, whereas SVM demonstrates superior performance by utilizing kernel functions to model intricate dependencies. Several studies have highlighted the effectiveness of support vector regression (SVR) in learning from historical process data to accurately predict crude oil properties. Ref. [150] predicted three product yields of the hydrodesulfurization (HDS) process, including the outlet sulphur concentration, percentage of SO_2_ emission, and percentage of biphenyl. In this study, a four-input SVR model hybridized with Bayesian optimization was employed, achieving a high degree of accuracy. The average experimental errors, the root mean square error (RMSE) and mean absolute error (MAE), were 0.022 and 0.097, respectively.

In the extended study of [148], the SVR model was optimized using a genetic algorithm to build models for HDS yield prediction. It was observed that the genetic algorithm is more accurate than the Bayesian algorithm for predicting HDS yield. This reflects the growing implementation of SVM models in modern process systems engineering, particularly under Industry 4.0 and digital refinery initiatives. Ref. [149] developed a smart SVR model to learn the relation between saturation pressure and crude oil composition data. Furthermore, five evolutionary algorithms were used to optimize SVR models. Among the different algorithms employed for optimizing SVR, the Bat-inspired algorithm (BA) exhibits better performance in the estimation of saturation pressure.

Alternatively, a deep learning technique was employed as a soft sensor in the petroleum refinery process/CDU to estimate and predict the online quality of the American Society for Testing and Materials (ASTM) 95% cut point temperature of heavy diesel [136]. The results were compared with those of other intelligent methods, including a single hidden layer neural network, SVM, PLS, and NNPLS. The study demonstrated that the deep learning-based network outperformed the other techniques in terms of RMSE and provided good generalization. SVM also exhibited a relatively low error compared to the single-layer neural network, PLS, and NNPLS, which exhibited slightly higher errors than deep learning. The SVM algorithm demonstrated strong predictive performance for product yield, supported by successful findings in the HDS process and CDU cut point temperature estimation, outperforming the partial least squares methods.

#### 6.2.3. Gradient Boosting

Gradient boosting (GB) algorithm models have gained extensive attention in prediction and correlation development, especially LightGBM and XGBoost. These models enhance prediction efficiency by comparing the predicted weak learner with the actual value for model learning, which is then applied to reduce error in subsequent training cycles.

One study used real-world sensor data from the Tüpras refinery and developed machine learning models using the GB algorithm to predict the C_5_ concentration in LPG and detect off-spec levels [147]. A voting regressor (VR) outperformed both a baseline median model and a linear regression approach, highlighting its superior ability to capture complex patterns in historical data. For off-spec detection, a CatBoost classifier trained with focal loss delivered the best performance (AUC ROC = 0.7670), proving its effectiveness in handling imbalanced data. Notably, incorporating data from an additional debutanizer unit significantly enhanced model accuracy, underscoring the value of comprehensive datasets in industrial ML applications. Furthermore, XGBoost has demonstrated superior performance in accurately predicting ethane and ethylene compositions in binary distillation columns [151], with high R^2^ values and low MAE and RMSE values. This demonstrates the potential of using XGBoost for other yield predictions with various manipulated parameters.

#### 6.2.4. Gaussian Process Regression

Machine learning also yields good predictions of the research octane number (RON) in the naphtha reforming and isomerization process [152]. In this study, the training process was observed using different ML models, such as SVM, GPR, ANN, regression tree (RT), and ensemble tree (ET). Training data was collected from Aspen HYSYS and MATLAB models through simulation of naphtha reforming and isomerization. The GPR, ANN, and SVM models outperformed the others, showing the highest prediction accuracy. GPR was chosen for SHapley Additive exPlanation (SHAP) to provide a comprehensive overview of the predictor contributing factors to the output. The study found that positive contributing factors are controlled by the reformer inlet temperature and feed flow rate, while the isomerization feed flow rate controls negative factors. Optimization of the GPR model using a genetic algorithm resulted in a 3.52% increase in the RON value. This explains that the GPR model can provide reasonable predictions and is suitable for reforming and isomerization processes.

## 7. Hybrid Approaches in Yield Estimation

Nowadays, oil refineries are realigning with the latest technology and AI. A few case studies have been conducted, providing a comprehensive overview of yield estimation that integrates laboratory, modelling, and simulation techniques for optimal performance. A summary of these hybrid approaches is tabulated in Table 5. A study on building an inferential estimation model for refinery feed oil classification was carried out to estimate the product flow rate and quality [153]. The atmospheric distillation column was simulated using HYSYS software, and the crude oil feed was varied by weight to produce different assay values for light, middle, and heavy components. To simulate real industrial situations, the MATLAB program facilitated data randomization for various operating conditions. The classification of crude oil was modelled using a bootstrap aggregated partial least squares (PLS) regression model by joining several neural networks. The input parameters for classification were the ratios of feed and product flow rates, and the modelling result was divided into several oil types: light, middle, and heavy components. Early prediction used a linear, single neural network and an aggregated network classifier. The findings showed that the aggregated network had the highest accuracy, whereas the linear classifier had the lowest. A comparison of single PLS and bootstrap-aggregated PLS showed higher classification accuracy and good robustness for both HYSYS-simulated and industrial data.

A study on the atmospheric distillation unit (ADU) was conducted to adopt a state-dependent parameter (SDP) as a soft sensor for product quality estimation and control [154]. The SDP is a data-driven model that provides good predictions for nonlinear systems, clearly representing the non-stationary characteristics within a simplified framework based on the system state or time. The study suggests a nonlinear SDP model in Equation (6), where *y*_*t*_ refers to model output, *n* is number of SDFs/regressors, *Z*_*i*,*t*_ is the i^*th*^ regressor, *a*_*i*,*t*_ is assumed to be constant, *a*_*i*_(.) is the SDP in the function of *i*^*th*^ correspondent state, *x*_*j*,*i*,*t*_, and *j* = 1, 2, … *ns*_*i*_. e_*t*_ is zero-mean white Gaussian-distributed unknown noise. If *a*_*i*,*t*_ is not state-dependent, *ns*_*i*_ is equal to 0.

(6) $$\left\{\begin{array}{c} y_{t}=\sum_{i=1}^{n}a_{i,t}\cdot z_{i,t}+e_{t}\\ a_{i,t}=a_{i}\left(x_{1,i,t},x_{2,i,t},\ldots ,x_{ns_{i},i,t}\right) \end{array},\;\forall t\right.$$

The SDP model can be estimated by applying various methods; however, this study used a polynomial concept that requires an instrumental variable (IV) to define the method: (1) for correlation with a regressor at the maximum extent, and (2) for correlation with an estimator at the least extent. The study introduced a combination of the IVs concept with polynomial modelling and fine-tuning of hyperparameters based on maximum likelihood (ML) approaches. The fine-tuned model executed prediction error decomposition (PED) using one-step-ahead predictions to restrain errors and develop an accurate model. For the ADU simulation, Aspen Dynamics and MATLAB-Simulink were incorporated, along with proportional integral plus (PIP) control, for the product set point limits (95% ASTM-D86, tray temperature) of naphtha, kerosene, and gas oil. The study concluded that the model prediction aligned greatly with the simulated data, with R^2^ values between 0.9784 and 0.9999 and an MAE below 0.5F for all three products. Besides, the adjusted R^2^ was equivalent to the actual value denoted by no integration of irrelevant variables. The implementation of the SDP model in a dynamic training environment demonstrated a rapid response, achieving simulated values within 2 h. A small distortion in the side product prediction time occurred; however, the output remained acceptable (<0.013% temperature differences) due to some overshoot and damping.

A study by [155] employed a combination of modelling and surrogate model-based optimization in the fluid catalytic cracking (FCC) process, which is a crucial unit process for converting heavy products. The study was categorized into three main sections, starting with hybrid data collection using real data and simulation, followed by multitask model learning for product yield estimation and optimization using surrogate models. The simulation dataset used Petro-SIM simulation, while the plant dataset was collected from control, execution, and information management systems. The collected data showed a concentrated data point for the plant data, while the simulation dataset exhibited a divergent distribution. The baseline model prediction results displayed excellent performance and accuracy in predicting plant data, with a low mean absolute percentage error (MAPE), compared to the simulation data, which illustrated a high level of distortion.

**Table 5:** Summary of hybrid approaches for crude oil yield estimation.

Hybrid Technique	Detailed Model	Key Findings	Limitations	Ref.
Simulation, mathematical modelling, and AI	HYSYS, MATLAB, inferential estimation, PLS (linear, single, aggregated network)	Bootstrap model estimates well across crude types and small datasets. Neural network enhances accuracy and robustness.	Requires classifier for crude oil before model integration. Crude oil changes significantly impact model generalization.	[153]
Simulation and mathematical modelling	HYSYS, MATLAB, SDF	Accurate prediction for nonlinear system. Fine-tuning reduces error and boosts accuracy. Fast response in dynamic environment.	Minor processing time deviation due to overshoot and damping. Requires more sensitive input than output variables.	[154]
Simulation and AI	Hybrid data (real data and simulation), PETRO-SIM, DNN and NLP optimization	Plant data enhance model prediction, performance, and accuracy. DNN improves simulation data accuracy. Model accuracy and efficiency depend on dataset quality.	Extrapolation causes inaccurate prediction. Requires good data quality. Significant feedstock changes lower performance and necessitate model retraining.	[155]
Laboratory, mathematical modelling, and AI	Spectroscopy, spectral pretreatment, PLS, ELM, RF	Mid-infrared spectroscopy shows high correlation coefficient. Suitable spectral pretreatment improves model optimization.	High dependency on spectral quality. Requires multiple spectral pretreatments.	[94]
Laboratory and AI	NMR, CNN, NNR, RVFL	NMR offers broad spectral range and high resolution for crude analysis. Deep learning offers better estimation. Deep learning offers greater accuracy and robustness than CNN model.	Requires transformation of spectral data into 2D. Requires pretraining process to mitigate overfitting issue.	[156]
Laboratory and mathematical modelling	ATR-IR and PLS model	Excellent prediction and model precision for well-blended crude oil. Provides qualitative and quantitative analysis. Reliable and simple prediction system.	Only suitable for pure crude oils. Less accurate for high-temperature yield. Requires good blending of crude oil. Encounters prediction inconsistency with non-crude oil blends.	[98]

The study proceeded with a deep neural network (DNN) that recorded the relationship between 14 inputs of properties and operating conditions and 6 outputs, consisting of the yield of petroleum products. The DNN consisted of two components: a parameter-sharing backbone with three layers of neurons to capture features from the dataset, and task-specific heads for output estimation. The prediction accuracy for the simulation data improved significantly, and the DNN model performed well on hybrid datasets. By leveraging a nonlinear programming (NLP) optimization model and Kernel Density Estimation (KDE), the yield of LNG, kerosene, and diesel increased with a relative difference in the range of +0.59%–+4.26%. The study concluded that the quality of the datasets influenced the model’s accuracy and prediction efficiency. The developed algorithm could provide a reasonable prediction with an error of less than 4.84% and 0.47% for the plant and simulation datasets, respectively.

A study on the application of spectroscopy and machine learning (ML) for prediction was conducted that used Raman spectroscopy and mid-infrared spectroscopy to quantify the kerosene content in gasoline [94]. The study integrated prediction models such as partial least squares (PLS), extreme learning machine (ELM), and random forest (RF) models with spectral pretreatment using Savitzky–Golay (SG), multiple scatter correction (MSC), standard normal variate (SNV), first-order, and second-order, which showed variation in their estimation. As the prediction process is highly dependent on spectral quality, pretreatment is crucial for the removal of interference and for noise cancellation. SG is specifically used for data smoothing, preserving information and removing noise; MSC is mostly used for data preprocessing, fine-tuning spectral data and data correlation; and SNV focuses on reducing the impact of particle size, surface scattering, and optical path changes.

The findings for mid-IR spectroscopy, expressed as SG-SNV pretreatment, yield the best effect for the PLS model (Rp = 0.9828), SNV hardlim for the ELM model (Rp = 0.9374), and SG-1st order for the RF model (Rp = 0.981), with the lowest root mean square error and the highest correlation coefficient. Meanwhile, for Raman spectroscopy, the ELM model yields the best prediction using SG-MSC (Rp = 0.9439), followed by the RF model with MSC only (Rp = 0.9634), and PLS remains similar to mid-IR spectroscopy, with SG-SNV (Rp = 0.7222). However, it is noticeable that the correlation coefficient for Raman spectroscopy is lower than that for mid-IR for all prediction models, suggesting that each model has different suitability in preprocessing spectra for optimized prediction. This study demonstrates that quantitative prediction can be successfully achieved through the use of a spectroscopy method and a simple pretreatment method for data tuning.

Another study presented in [156] utilizes nuclear magnetic resonance (NMR) spectroscopy combined with a deep learning soft sensor, a nearest neighbour learning part, and a random vector functional link (RVFL) network, along with spectral data preprocessing, to evaluate yield fractions. Similarly to in previous studies, it is demonstrated that data preprocessing can transform spectral data, produce virtual samples, and improve data replicability. NMR spectroscopy offers good spectral resolution and a broad measurement range in determining the physicochemical properties of crude oil. The proposed model introduces a convolution neural network (CNN) and near neighbour regression (NNR) as the component learner, followed by RVFL as a meta learner to incorporate both component learners. Spectral data from NMR require alteration into 2D format for CNN application. Furthermore, the pretraining process and training times cause differences in modelling time and are needed in order to reduce overfitting issues. The results show that single-ensemble deep learning yields better predictions than the CNN model, while multiple ensembles significantly improve precision. The multiple ensemble deep learning soft sensor model offers excellent accuracy and robustness for yield prediction using NMR.

The integration of attenuated total reflection IR spectra (ATR-IR) with the PLS model shows good agreement in prediction of the yield of crude blends on the condition that pure crude oils are included [98]. For non-crude oil feedstocks, such as residues, the prediction deviates and is less accurate due to the dominance of high-temperature yields. These findings suggest that efficient blending of crude oil is crucial for accurate prediction. The computed yield from the database of crude assay and volume percentage yields similar results to those of ATR prediction, suggesting fluctuations in prediction for non-crude oil blends. However, the proposed PLS model with ATR-IR provides good prediction and excellent accuracy for crude oil yield blends. ATR-IR spectroscopy can provide both qualitative and quantitative analysis of crude oil using a reliable, less complicated system, as well as highlighting variety of crude oil properties.

The hybrid technique offers outstanding prediction with high accuracy, reliability, and a fast response in crude oil yield estimation. Detailed information on crude oil properties from lab work and simulation, combined with automated prediction, allows for good pattern learning, which simultaneously enhances the final prediction. The integration of hybrid approaches can significantly improve yield estimation and directly facilitate operational management in an oil refinery.

## 8. Future Prospects

To enhance the accuracy of crude oil yield estimation, future exploration should aim for the integration of more advanced technologies and methodologies.

- Hybrid approaches exhibit good predictive ability by implementing multiple methods in data collection and modelling. Thus, future work should incorporate diverse sources of data collection in the development of a synchronized model with outstanding prediction accuracy.
- Prediction efficiency is significantly impacted by the data size used for data learning, resulting in lower bias, reduced overfitting, and a deeper understanding of more complex relationships. It is suggested to improve the data bank through data storage and the creation of a centralized platform for a variety of data collection tasks, including cloud-based and data science applications.
- As data-driven approaches continue to positively improve the prediction of crude oil, enhancing model development is the best option. Incorporating automation into data preprocessing can reduce fluctuation in estimation with higher accuracy and precision.
- A dynamic yield forecast can be achieved through the use of soft sensors and real-time monitoring. Continuous data prediction is helpful in decision-making and optimizing plant operations for a sustainable future.

## 9. Conclusions

In conclusion, estimation of the yield of petroleum products is essential for understanding the properties of crude oil, as it provides an initial overview of the product fractions. The selection of appropriate estimation techniques, ranging from traditional modelling to AI-driven methods, exhibits both benefits and drawbacks specific to their nature. Traditional approaches provide a detailed, thorough, and direct analysis; meanwhile, advanced methods improve the precision and accuracy of the prediction. With the latest technological advancements, AI has been widely integrated into various fields and applications for real-time monitoring, fast response, and high consistency. The hybridization and integration of multiple methods offer ultimate potential and benefits for the future oil and gas industry. Future work should emphasize the implementation of efficient techniques with high reliability, accuracy, and sustainability to address the challenges in the forthcoming state of the energy sector.

## Figures and Tables

**Table 1:** Summary of physical distillation (ASTM D-2892) and simulated distillation (SIMDIS) [82].

Key Attributes	Physical Distillation (ASTM D-2892)	Simulated Distillation (SIMDIS)
Methodology	Light distillate: ASTM D-2892 with 15 theoretical plate and reflux of 5:1. Heavy distillate: ASTM D-5236.	Using gas chromatography with an FID detector. Software for SIMDIS calculation method.
Advantages	Detailed and direct measurement. Maintain quality standard.	Enhanced confidence level with minimal error. Efficient and consistent detection. Low chance of accidents and contamination.
Disadvantages	Excessively time-consuming. Extensive operator involvement.	Detection issues in high-boiling-point scenarios. Instrument sensitivity.

**Table 2:** Simulation tools for yield estimation.

Simulation Software	Usage	Strength	Limitation	Ref.
AspenTech: Version v.8.8, v7.1 and v.11	Software simulation in chemical, petrochemical, energy, and oil and gas industries	Includes economic and environmental analysis. Reliable and widely applied in industrial and research fields. Well-integrated platform and compatible system for complex model.	Complexity operational simulation. Sensitive in steady state to dynamic environment transition.	[103,104,105]
PETRO-SIM	Process simulation in petrochemical industry and oil and gas refining	Enables efficient performance management and rapid prototyping. Provides action list for optimization. Concerns regarding real-time application.	Less functionality for dynamic simulation.	[106,107]
UniSim Simulator	Process simulation in petrochemical industry and oil and gas refining	Enables automatic control systems. Supports operation training control and dynamic simulation. Provides automated calculation.	Not extensively used.	[108,109]

**Table 3:** Comparison of mathematical modelling and machine learning.

Key Point	Mathematical Modelling	Machine Learning
Definition	Represents real-world scenarios for data analysis consideration.	Represents the pattern of input data for the prediction.
Methodology	Apply mathematical terms and a kinetics equation.	Develop an algorithm and statistical model to allow the formation of data patterns.
Data source	Experimental and lab data.	Historical data.
End target	Mathematical equation.	Prediction model.

## Data Availability

Data is contained within the article.

## References

[1] Alsayoof L., Shams M.B. (2022). The role of crude oil selection in enhancing the profitability of a local refinery with lube hydro-processing capacity. Chem. Eng. Res. Des..

[2] Stratiev D., Dinkov R., Nikolaev N., Stanulov K. (2010). Evaluation of impact of crude oil quality on refinery profit. Erdoel Erdgas Kohle.

[3] Wu J.X., Li S.F., Li Q.F., Yan F., Zhou Q.L., Ma S., Zhang Y.H., Zhao S.Q., Shi Q. (2024). Characterization of chemical composition of high viscosity heavy oils: Macroscopic properties, and semi-quantitative analysis of molecular composition using high-resolution mass spectrometry. Pet. Sci..

[4] Sivasakthi A., Nagalakshmi T. (2018). Characterization of heavy crude oil through physical and chemical properties. Int. J. Sci. Adv. Res. Technol. IJSART.

[5] Marfo S.A., Appau P.O., Tettegah D. (2019). Software approach on crude oil yields prediction: A case study of Tema Oil Refinery. J. Petroleum. Gas Eng..

[6] Koçman Ö., Atay Ö., Zehir C. (2025). Implementing Green Management in the Petroleum Industry: A Model Proposal for Türkiye. Energies.

[7] Malaysian Investment Development Authority (2025). (MIDA) Oil and Gas Industry in Malaysia.

[8] Devold H. (2013). Oil and Gas Production Handbook: An Introduction to Oil and Gas Production.

[9] Álvarez E., Bravo M., Jiménez B., Mourão A., Schultes R. (2018). The Oil and Gas Value Chain: A Focus on Oil Refining.

[10] Darko E. (2014). Short Guide Summarising the Oil and Gas Industry Lifecycle for a Non-Technical Audience.

[11] Craig J., Quagliaroli F. (2020). The oil & gas upstream cycle: Exploration activity. Proceedings of the EPJ Web of Conferences.

[12] Hadzihafizovic D. (2024). Petroleum Refining Processes.

[13] Puhan D., Casford M.T., Davies P.B. (2023). Evaluation of structural and compositional changes of a model monoaromatic hydrocarbon in a Benchtop hydrocracker using GC, FTIR, and NMR spectroscopy. ACS Omega.

[14] Cerić E. (2012). Crude Oil, Processes and Products.

[15] Speight J.G. (2016). Handbook of Petroleum Refining.

[16] Eldos H.I., Khan M., Zouari N., Saeed S., Al-Ghouti M.A. (2022). Characterization and assessment of process water from oil and gas production: A case study of process wastewater in Qatar. Case Stud. Chem. Environ. Eng..

[17] Adiko S.B., Mingasov R.R. (2020). Crude Distillation Unit (CDU). Analytical Chemistry-Advancement, Perspectives and Applications.

[18] Pak A., Mohammadi T. (2008). Wastewater treatment of desalting units. Desalination.

[19] Kundnaney N.D., Kushwaha D.K. A critical review on heat exchangers used in oil refinery. Proceedings of the 3rd Afro-Asian International Conference on Science, Engineering and Technology.

[20] Olsen T. (2014). An oil refinery walk-through. Chem. Eng. Prog..

[21] Speight J.G. (2020). The Refinery of the Future.

[22] Thirunavukkarasu I., Janani R., Yadav E.S., Selvanathan S.P. Modeling and control of tray temperature along with column pressure in a pilot plant distillation column. Proceedings of the 2019 International Conference on Automation, Computational and Technology Management (ICACTM).

[23] Liu Z.Y., Jobson M. (1999). The effect of operating pressure on distillation column throughput. Comput. Chem. Eng..

[24] Speight J.G. (2023). Thermal and Catalytic Processing in Petroleum Refining Operations.

[25] Cheremisinoff N.P., Rosenfeld P.E. (2009). Handbook of Pollution Prevention and Cleaner Production Volume 1: Best Practices in the Petroleum Industry.

[26] Speight J.G. (2019). Heavy Oil Recovery and Upgrading.

[27] Akhtar F., Andersson L., Ogunwumi S., Hedin N., Bergström L. (2014). Structuring adsorbents and catalysts by processing of porous powders. J. Eur. Ceram. Soc..

[28] Foong S.Y., Chan Y.H., Cheah W.Y., Kamaludin N.H., Ibrahim T.N.B.T., Sonne C., Peng W., Show P.L., Lam S.S. (2021). Progress in waste valorization using advanced pyrolysis techniques for hydrogen and gaseous fuel production. Bioresour. Technol..

[29] Sampson I., Harcourt P. Catalytic Polymerisation of Light Gases Majorly Olefins to Produce Polymer Gasoline. Proceedings of the 44TH Annual Conference of Nigerian Society of Chemical.

[30] Speight J.G., El-Gendy N.S. (2017). Introduction to Petroleum Biotechnology.

[31] Shipman R. (2002). Liquefied petroleum gas. Plant Engineer’s Reference Book.

[32] Civan F., Cleveland C. (2004). Natural Gas Transportation and Storage. Encycl. Energy.

[33] Synák F., Čulík K., Rievaj V., Gaňa J. (2019). Liquefied petroleum gas as an alternative fuel. Transp. Res. Procedia.

[34] Hammeed G., Orifah M., Ijeoma M., Tijani S. (2016). Assessment of the Use of Liquefied Petroleum Gas (LPG) as Cooking Energy Source Among Rural Households in Badagry Area of Lagos State. Am. Sci. Res. J. Eng. Technol. Sci. (ASRJETS).

[35] Abbas F.A. (2022). LPG as an Alternative Fuel of Automobile in Iraq.

[36] Silva A.P., Bahú J.O., Soccol R., Rodríguez-Urrego L., Fajardo-Moreno W.S., Moya H., León-Pulido J., Cárdenas Concha V.O. (2023). Naphtha characterization (PIONA, density, distillation curve and sulfur content): An origin comparison. Energies.

[37] Speight J.G. (2019). Handbook of Industrial Hydrocarbon Processes.

[38] Redwan D.S., Abu-Shbak M.M., Bubshait K.A. (1999). Supply and demand of light naphtha as potential petrochemical feedstock in Saudi Arabia. Pet. Sci. Technol..

[39] Badra J., Elwardany A., Sim J., Viollet Y., Im H.G., Chang J. Effects of in-cylinder mixing on low octane gasoline compression ignition combustion. Proceedings of the SAE 2016 World Congress and Exhibition.

[40] Magaril E., Magaril R. (2020). Application of modified gasoline to increase energy efficiency and environmental parameters of vehicle operation. WIT Trans. Ecol. Environ..

[41] Outlook W.F. (2010). Short-Term Energy Outlook.

[42] Speight J. (2011). Production, properties and environmental impact of hydrocarbon fuel conversion. Advances in Clean Hydrocarbon Fuel Processing.

[43] Speight J.G. (2015). Handbook of Petroleum Product Analysis.

[44] Mazurek W., Kemp T., Bruce G., Forrest J. Understanding the Impact of Refined Product Properties on Synthetic Crude Oil and Bitumen Marketability. Proceedings of the PETSOC 6th Canadian International Petroleum Conference.

[45] Kittel H., Straka P., Šimáček P., Kadleček D. (2023). Kerosene from hydrocracking for JET fuel with reduced aromatic content. Pet. Sci. Technol..

[46] Lam N.L., Smith K.R., Gauthier A., Bates M.N. (2012). Kerosene: A review of household uses and their hazards in low-and middle-income countries. J. Toxicol. Environ. Health Part B.

[47] Aitani A.M. (2004). Oil Refining and Products. Encyclopedia of Energy.

[48] Demirbas A., Acar S., Horasan B.Y., Alalayah W.M. (2018). Analysis of petroleum coke from low grade oily sludge of refinery. Pet. Sci. Technol..

[49] Wang S. (2024). The Application of Diesel Engine: From 21st Century to Modern Life. Highlights Sci. Eng. Technol..

[50] Marketing G. (2007). Diesel Fuels Technical Review.

[51] Bart J.C., Gucciardi E., Cavallaro S. (2012). Biolubricants: Science and Technology.

[52] Zhang C., Li K., Luo J. (2021). Superlubricity with nonaqueous liquid. Superlubricity.

[53] Khudhur A.G., Mohammed Z.I. (2020). Statistical Model for Re-Refining of Used Lubricating Oil by Solvent Extraction and Bentonite Clay Adsorption Method. Proceedings of the IOP Conference Series: Materials Science and Engineering.

[54] Ait Allal A., Mansouri K., Youssfi M., Qbadou M. (2019). Toward an evaluation of marine fuels for a clean and efficient autonomous ship propulsion energy. Mater. Today Proc..

[55] IARC Working Group on the Evaluation of Carcinogenic Risks to Humans (1989). FUEL OILS (HEATING OIL). Occupational Exposures in Petroleum Refining.

[56] Vermeire M.B. (2021). Everything You Need to Know About Marine Fuels.

[57] Speight J.G. (2016). Chapter 8-Uses of Asphalt.

[58] Zhang H. (2019). Introductory chapter: Asphalt and asphalt mixture. Asphalt and Asphalt Mixtures.

[59] Shestakov N., Putilin S. (2018). Application of water-organic emulsions for the recovery of asphalt concrete. Proceedings of the MATEC Web of Conferences.

[60] Tillman D.A., Duong D.N., Harding N.S. (2012). Solid Fuel Blending.

[61] Boateng A.A. (2015). Rotary Kilns: Transport Phenomena and Transport Processes.

[62] Parraga J., Khalilpour K.R., Vassallo A. (2018). Polygeneration with biomass-integrated gasification combined cycle process: Review and prospective. Renew. Sustain. Energy Rev..

[63] Ibrahim H.A.H. (2005). The effect of thermal treatment on the true density of Syrian green delayed petroleum coke. Arab. J. Sci. Eng..

[64] Shishkova I., Stratiev D., Kolev I.V., Nenov S., Nedanovski D., Atanassov K., Ivanov V., Ribagin S. (2022). Challenges in petroleum characterization—A review. Energies.

[65] Wauquier J.P. (1995). Petroleum Refining: Crude Oil, Petroleum Products, Process Flowsheets.

[66] Haglund P.S., Löfstrand K., Siek K., Asplund L. (2013). Powerful GC-TOF-MS techniques for screening, identification and quantification of halogenated natural products. Mass Spectrom..

[67] Aidha N.N., Yunilawati R., Rumondang I. Method development for analysis of essential oils authenticity using gas chromatography-mass spectrometry (GC-MS). Proceedings of the 2nd International Conference of Essential Oil Indonesia (ICEO).

[68] Chua C.C., Brunswick P., Kwok H., Yan J., Cuthbertson D., van Aggelen G., Helbing C.C., Shang D. (2020). Enhanced analysis of weathered crude oils by gas chromatography-flame ionization detection, gas chromatography-mass spectrometry diagnostic ratios, and multivariate statistics. J. Chromatogr. A.

[69] Reddy C.M., Quinn J.G. (1999). GC-MS analysis of total petroleum hydrocarbons and polycyclic aromatic hydrocarbons in seawater samples after the North Cape oil spill. Mar. Pollut. Bull..

[70] Feng T., Sun M., Song S., Zhuang H., Yao L. (2019). Gas chromatography for food quality evaluation. Evaluation Technologies for Food Quality.

[71] Sudhakar P., Latha P., Reddy P. (2016). Phenotyping Crop Plants for Physiological and Biochemical Traits.

[72] Gebruers K., Courtin C.M., Delcour J.A. (2009). Quantification of arabinoxylans and their degree of branching using gas chromatography. Healthgrain Methods: Analysis of Bioactive Components in Small Grain Cereals.

[73] Briker Y., Ring Z., Iacchelli A., McLean N., Rahimi P., Fairbridge C., Malhotra R., Coggiola M., Young S. (2001). Diesel fuel analysis by GC- FIMS: Aromatics, n-paraffins, and isoparaffins. Energy Fuels.

[74] Malhotra R., Coggiola M., Young S., Hsu C., Dechert G., Rahimi P. (1998). Rapid detailed analysis of diesel fuels by GC-FIMS: Chemistry of diesel fuels. Prepr.-Am. Chem. Soc. Div. Pet. Chem..

[75] Ogawa T. (2005). Analytical conditions for field ionization mass spectrometry of diesel fuel. Fuel.

[76] Dallüge J., van Rijn M., Beens J., Vreuls R.J., Brinkman U.A.T. (2002). Comprehensive two-dimensional gas chromatography with time-of-flight mass spectrometric detection applied to the determination of pesticides in food extracts. J. Chromatogr. A.

[77] Mao F., Wang J., Fan H. (2021). Application of two-dimensional gas chromatography/time-of-flight mass spectrometry (GC× GC-TOFMS) for the thorough study of hydrocarbons in lignite pyrolysates. J. Anal. Appl. Pyrolysis.

[78] Thurbide K.B., Cooke B.W., Aue W.A. (2004). Novel flame photometric detector for gas chromatography based on counter-current gas flows. J. Chromatogr. A.

[79] Hinshaw J.V. (2018). A Compendium of GC Detection, Past and Present.

[80] Rodrigues É.V., Silva S.R., Romão W., Castro E.V., Filgueiras P.R. (2018). Determination of crude oil physicochemical properties by high-temperature gas chromatography associated with multivariate calibration. Fuel.

[81] Lopes M., Lopes M.S., Maciel Filho R., Maciel M.W., Medina L. (2012). Extension of the TBP curve of petroleum using the correlation DESTMOL. Procedia Eng..

[82] Espinosa-Peña M., Figueroa-Gómez Y., Jiménez-Cruz F. (2004). Simulated distillation yield curves in heavy crude oils: A comparison of precision between ASTM D-5307 and ASTM D-2892 physical distillation. Energy Fuels.

[83] (2020). Standard Test Method for Distillation of Crude Petroleum (15-Theoretical Plate Column).

[84] (2018). Standard Test Method for Distillation of Heavy Hydrocarbon Mixtures (Vacuum Potstill Method).

[85] Pasadakis N., Xekoukoulotakis N. (2007). Gas chromatographic analysis of crude oils with thermal extraction sampling. Pet. Sci. Technol..

[86] Ha H.Z., Ring Z., Liu S. (2008). Data reconciliation among PIONA, GC-FIMS, and SimDis measurements for petroleum fractions. Pet. Sci. Technol..

[87] Lissitsyna K., Huertas S., Quintero L., Polo L. (2014). PIONA analysis of kerosene by comprehensive two-dimensional gas chromatography coupled to time of flight mass spectrometry. Fuel.

[88] Chen G., Zhou Z., Chen Y., Zhao X., Han Q., Yin X., Zang Y. (2020). Evaluation of Crude Oil Rheology as a Comprehensive Experimental for the Applied Chemistry Education. Proceedings of the International Conference on Arts, Humanity and Economics, Management (ICAHEM 2019).

[89] National Academies of Sciences, Engineering, and Medicine, Division on Earth and Life Studies, Board on Chemical Sciences and Technology, Committee on the Effects of Diluted Bitumen on the Environment (2016). Spills of Diluted Bitumen from Pipelines: A Comparative Study of Environmental Fate, Effects, and Response.

[90] Santos I., Oliveira P., Mansur C. (2017). Factors that affect crude oil viscosity and techniques to reduce it: A review. Braz. J. Pet. Gas.

[91] Chen G., Zhu X., Jia K., Li Y., Zhu L. (2021). Sulfur Analysis and Sulfur Transfer Rule During Simulated Thermal Processing of Heavy Oil. Proceedings of the IOP Conference Series: Earth and Environmental Science.

[92] Yang S.B., Moreira J., Li Z. (2022). Predicting crude oil properties using fourier-transform infrared spectroscopy (FTIR) and data-driven methods. Digit. Chem. Eng..

[93] Abdulkadir I., Uba S., Almustapha M. (2016). A rapid method of crude oil analysis using FT-IR spectroscopy. Niger. J. Basic Appl. Sci..

[94] Li X., Liu Y., Jiang X., Ouyang A., Sun X., Wang G. (2020). Determination and quantification of kerosene in gasoline by mid-infrared and Raman spectroscopy. J. Mol. Struct..

[95] Peng S., Ye C., Liu M. (2003). Quantitative Estimation of Property Parameters of Crude Oil Using Two-Dimensional 13C–1H J-resolved Nuclear Magnetic Resonance Spectroscopy (HET-JRES). Appl. Spectrosc..

[96] Steffens J., Landulfo E., Courrol L.C., Guardani R. (2011). Application of fluorescence to the study of crude petroleum. J. Fluoresc..

[97] Ryder A.G. (2005). Analysis of crude petroleum oils using fluorescence spectroscopy. Reviews in Fluorescence 2005.

[98] Šašić S., Grant C., Mize R., Patel D., Nolte J., Haendel R., Grüner C., van Wezel R. (2025). Predicting yields in crude oil blends via multivariate modelling of crude oil ATR/IR spectra. Microchem. J..

[99] Masili A., Puligheddu S., Sassu L., Scano P., Lai A. (2012). Prediction of physical–chemical properties of crude oils by 1H NMR analysis of neat samples and chemometrics. Magn. Reson. Chem..

[100] Kök M.V., Varfolomeev M.A., Nurgaliev D.K. (2019). Determination of SARA fractions of crude oils by NMR technique. J. Pet. Sci. Eng..

[101] Yu J. Prediction of the Composition and Proportion of Blended Crude Oil Using Near-Infrared Spectroscopy. Proceedings of the 2023 2nd International Conference on Advanced Sensing, Intelligent Manufacturing (ASIM).

[102] Patrascioiu C., Stamatescu G. (2015). Petroleum Fractions Liquid–Vapor Equilibrium Simulation using Unisim Design. Rev. Chim..

[103] Bartolome P.S., Van Gerven T. (2022). A comparative study on Aspen Hysys interconnection methodologies. Comput. Chem. Eng..

[104] Olugbenga A.G., Al-Mhanna N.M., Yahya M.D., Afolabi E.A., Ola M.K. (2021). Validation of the molar flow rates of oil and gas in three-phase separators using Aspen Hysys. Processes.

[105] Vasudevan S., Konda N.M., Zhang C. (2012). Potential Problems with Rigorous Simulators and Possible Solutions. Reviews in Fluorescence 2005.

[106] Aylott M., Van der Merwe B. Petro-SIM simulator and cape-open: Experiences and successes. Proceedings of the 2008 AIChE Annual Meeting.

[107] Sayles S., Routt D.M. (2011). Unconventional crude oil selection and compatibility. Digit. Refin..

[108] Foo D. (2022). Chemical Engineering Process Simulation.

[109] Patrascioiu C., Popescu M., Paraschiv N. (2014). Specific Problems of Using Unisim Design in the Dynamic Simulation of the Propylene-Propane Distillation Column. Rev. Chim..

[110] AspenTech Over 40 Years of Innovation, 2025. https://www.aspentech.com/en/about-aspentech/history.

[111] Rezaie Azizabadi H., Ziabasharhagh M., Mafi M. (2021). Applicability of the common equations of state for modeling hydrogen liquefaction processes in Aspen HYSYS. Gas Process. J..

[112] Sarpong-Mensah J. (2023). Crude Oil Distillation Using Aspen Hysys. Problem Solving Exercise. Module 701004 Petroleum and Petrochemical Engineering.

[113] Almansouri H.E.O. (2022). Simulation of Sarir Crude Oil Refinery Using Aspen Hysys. J. Eng. Res..

[114] (2023). Standard Test Method for Distillation of Petroleum Products and Liquid Fuels at Atmospheric Pressure.

[115] Kamışlı F., Ahmed A.A. (2019). Simulation and Optimization of A Crude Oil Distillation Unit. Turk. J. Sci. Technol..

[116] Jaja Z., Akpa J.G., Dagde K.K. (2020). Optimization of crude distillation unit case study of the Port Harcourt Refining Company. Adv. Chem. Eng. Sci..

[117] Parthiban N., Nagarajan N., Mahendra V., Senthil K. (2013). Dynamic modeling and simulation of crude fractionation column with three side strippers using Aspen HYSYS Dynamics: A best practice for crude distillation column dynamic modeling. J. Pet. Gas Explor. Res..

[118] Petro-SIM 7.2 Changes to Refinery Reactors. https://www.petro-sim.com/php/pmwiki/pmwiki.php?n=Build72.ChangeListRX.

[119] Lopez-Rodriguez A., Arteagabeitia A., Martinez-Camara J.M., Aguilar C., Jimenez-Asenjo P. Rigorous refinery-wide optimisation: A case study for Petronor. Proceedings of the 19th World Petroleum Congress.

[120] AL-Dogail A., Gajbhiye R., Al-Shammari H., Alnaser M., Kamerkar T. (2023). Maximization of Gas-Oil Separation Plant Oil Recovery by Operation Parameter Optimization. SPE Prod. Oper..

[121] Kavousi K., Mokhtarian N. (2015). Simulation the continuous catalytic reforming (octanizer) unit of Isfahan refinery gasoline production complex with PETROSIM software. Int. Sci. Investig. J..

[122] Patrascioiu C., Jamali M. (2018). Crude distillation process simulation using Unisim Design simulator. Int. J. Chem. Mol. Eng..

[123] Patrascioiu C. (2019). Modelling the Atmospheric Distillation Using the Unisim Design Simulator. Proceedings of the MATEC Web of Conferences.

[124] Rahman S.A., Anjana R. (2021). Unisim Based Simulation and Analysis of Crude Oil Distillation. Proceedings of the IOP Conference Series: Materials Science and Engineering.

[125] Wang X.G., Yang Y.Y. (2012). Simulation of Reactive Distillation Process with ChemCAD Software. Adv. Mater. Res..

[126] Galusnyak S.C., Petrescu L., Cormos C.C. (2022). Classical vs. reactive distillation technologies for biodiesel production: An environmental comparison using LCA methodology. Renew. Energy.

[127] Lei Q., Munir M.T., Bao J., Young B. (2016). A data-driven fault detection method based on dissipative trajectories. IFAC-PapersOnLine.

[128] Tangsriwong K., Lapchit P., Kittijungjit T., Klamrassamee T., Sukjai Y., Laoonual Y. (2020). Modeling of chemical processes using commercial and open-source software: A comparison between Aspen Plus and DWSIM. Proceedings of the IOP Conference Series: Earth and Environmental Science.

[129] Jain R., Nayak P., Rahul A.S., Dalve P., Moudgalya K.M., Naren P., Wagner D., Fritzson P. (2019). Implementation of a property database and thermodynamic calculations in openmodelica for chemical process simulation. Ind. Eng. Chem. Res..

[130] ChemSep (2023). ChemSep—Equilibrium Column Simulator. http://www.chemsep.com/.

[131] Skorych V., Dosta M., Heinrich S. (2020). Dyssol—An open-source flowsheet simulation framework for particulate materials. SoftwareX.

[132] Moliner C., Marchelli F., Bosio B., Arato E. (2018). Simulation of the gasification of agricultural residues using coco simulator. Proceedings of the EUBCE 2018 Conference Proceedings.

[133] Giordano G.F., Vieira L.C., Gomes A.O., de Carvalho R.M., Kubota L.T., Fazzio A., Schleder G.R., Gobbi A.L., Lima R.S. (2021). Distilling small volumes of crude oil. Fuel.

[134] Ahmadlouydarab M., Hosseini S.S., Muhammad Ali H., Asadzadeh N. (2023). A Precise Mathematical Correlation to Estimate Product Yield of Delayed Coking Units. J. Chem. Pet. Eng..

[135] Qunyong L., Qingyin J., Zhikai C., Lijiang A., Yuqin Z. Modeling and simulation for the hydrocracking reactor. Proceedings of the 2008 27th Chinese Control Conference.

[136] Shang C., Yang F., Huang D., Lyu W. (2014). Data-driven soft sensor development based on deep learning technique. J. Process Control.

[137] Kwon H., Oh K.C., Choi Y., Chung Y.G., Kim J. (2021). Development and application of machine learning-based prediction model for distillation column. Int. J. Intell. Syst..

[138] Biyanto T.R., Suhartanto T., Widjiantoro B.L. (2007). Predicting Liquid-Vapor (LV) composition at distillation column. Songklanakarin J. Sci. Technol..

[139] Singh V., Gupta I., Gupta H. (2005). ANN based estimator for distillation—Inferential control. Chem. Eng. Process. Process Intensif..

[140] Rogina A., Šiško I., Mohler I., Ujević Ž., Bolf N. (2011). Soft sensor for continuous product quality estimation (in crude distillation unit). Chem. Eng. Res. Des..

[141] Fortuna L., Graziani S., Xibilia M.G. (2005). Soft sensors for product quality monitoring in debutanizer distillation columns. Control Eng. Pract..

[142] De Canete J.F., del Saz-Orozco P., Gonzalez S., García-Moral I. (2012). Dual composition control and soft estimation for a pilot distillation column using a neurogenetic design. Comput. Chem. Eng..

[143] Bahar A., Özgen C. (2010). State estimation and inferential control for a reactive batch distillation column. Eng. Appl. Artif. Intell..

[144] Fileti A.M.F., Cruz S.L., Pereira J.A. (2000). Control strategies analysis for a batch distillation column with experimental testing. Chem. Eng. Process. Process Intensif..

[145] Zamprogna E., Barolo M., Seborg D. (2001). Composition estimations in a middle-vessel batch distillation column using artificial neural networks. Chem. Eng. Res. Des..

[146] Singh V., Gupta I., Gupta H. (2007). ANN-based estimator for distillation using Levenberg–Marquardt approach. Eng. Appl. Artif. Intell..

[147] Rožanec J.M., Trajkova E., Lu J., Sarantinoudis N., Arampatzis G., Eirinakis P., Mourtos I., Onat M.K., Yilmaz D.A., Košmerlj A. (2021). Cyber-physical lpg debutanizer distillation columns: Machine-learning-based soft sensors for product quality monitoring. Appl. Sci..

[148] Al-Jamimi H.A., BinMakhashen G.M., Saleh T.A. (2022). Artificial intelligence approach for modeling petroleum refinery catalytic desulfurization process. Neural Comput. Appl..

[149] Al-Jamimi H.A., BinMakhashen G.M., Saleh T.A. (2022). Multiobjectives optimization in petroleum refinery catalytic desulfurization using Machine learning approach. Fuel.

[150] Ansari H.R., Gholami A. (2015). An improved support vector regression model for estimation of saturation pressure of crude oils. Fluid Phase Equilibria.

[151] Pullanikkattil S., Yerolla R., Besta C.S. (2025). Interpretable Machine learning model for predicting Ethane-Ethylene composition in binary distillation process. Therm. Sci. Eng. Prog..

[152] Saghir H., Ahmad I., Kano M., Caliskan H., Hong H. (2024). Prediction and optimisation of gasoline quality in petroleum refining: The use of machine learning model as a surrogate in optimisation framework. CAAI Trans. Intell. Technol..

[153] Zhou C., Liu Q., Huang D., Zhang J. (2012). Inferential estimation of kerosene dry point in refineries with varying crudes. J. Process Control.

[154] Bidar B., Khalilipour M.M., Shahraki F., Sadeghi J. (2018). A data-driven soft-sensor for monitoring ASTM-D86 of CDU side products using local instrumental variable (LIV) technique. J. Taiwan Inst. Chem. Eng..

[155] Li H., Zhao Q., Wang R., Xu W., Qiu T. (2024). Integrated Hybrid Modelling and Surrogate Model-Based Operation Optimization of Fluid Catalytic Cracking Process. Processes.

[156] Yi L., Lu J., Ding J., Liu C., Chai T. (2020). Soft sensor modeling for fraction yield of crude oil based on ensemble deep learning. Chemom. Intell. Lab. Syst..

